# Anti-Inflammatory and Pro-Autophagy Effects of the Cannabinoid Receptor CB2R: Possibility of Modulation in Type 1 Diabetes

**DOI:** 10.3389/fphar.2021.809965

**Published:** 2022-01-18

**Authors:** Qing-Rong Liu, Kanikkai Raja Aseer, Qin Yao, Xiaoming Zhong, Paritosh Ghosh, Jennifer F. O’Connell, Josephine M. Egan

**Affiliations:** ^1^ Laboratory of Clinical Investigation, National Institute on Aging, NIH, Baltimore, MD, United States; ^2^ Ben May Department for Cancer Research, The University of Chicago, Chicago, IL, United States

**Keywords:** type 1 diabetes mellitus, cannabinoid receptor, autoimmunity, autophagy, inflammation, immunetolerance, lysosome

## Abstract

Type 1 diabetes mellitus (T1DM) is an autoimmune disease resulting from loss of insulin-secreting β-cells in islets of Langerhans. The loss of β-cells is initiated when self-tolerance to β-cell-derived contents breaks down, which leads to T cell-mediated β-cell damage and, ultimately, β-cell apoptosis. Many investigations have demonstrated the positive effects of antagonizing cannabinoid receptor 1 (CB1R) in metabolic diseases such as fatty liver disease, obesity, and diabetes mellitus, but the role of cannabinoid receptor 2 (CB2R) in such diseases is relatively unknown. Activation of CB2R is known for its immunosuppressive roles in multiple sclerosis, rheumatoid arthritis, Crohn’s, celiac, and lupus diseases, and since autoimmune diseases can share common environmental and genetic factors, we propose CB2R specific agonists may also serve as disease modifiers in diabetes mellitus. The *CNR2* gene, which encodes CB2R protein, is the result of a gene duplication of *CNR1*, which encodes CB1R protein. This ortholog evolved rapidly after transitioning from invertebrates to vertebrate hundreds of million years ago. Human specific *CNR2* isoforms are induced by inflammation in pancreatic islets, and a *CNR2* nonsynonymous SNP (Q63R) is associated with autoimmune diseases. We collected evidence from the literature and from our own studies demonstrating that CB2R is involved in regulating the inflammasome and especially release of the cytokine interleukin 1B (IL-1β). Furthermore, CB2R activation controls intracellular autophagy and may regulate secretion of extracellular vesicles from adipocytes that participate in recycling of lipid droplets, dysregulation of which induces chronic inflammation and obesity. CB2R activation may play a similar role in islets of Langerhans. Here, we will discuss future strategies to unravel what roles, if any, CB2R modifiers potentially play in T1DM.

## Introduction

### Overview of Type 1 Diabetes Mellitus and its Etiology

Based on the 2020 CDC’s National Diabetes Statistics Report, the number of people in the United States suffering from type 1 diabetes mellitus (T1DM) increased from 1.25 to 1.6 million between 2017 and 2020 (https://www.cdc.gov/diabetes/data/statistics-report/index.html). In addition to the increasing incidence, the peak age at diagnoses has shifted to an even younger age group (Ilonen et al., 2019). Overall, the highest incidence of T1DM is in Northern European countries and the island of Sardinia, while lower incidences are reported from India and China (Patterson et al., 2019). The underlying mechanism of pancreatic β-cell failure involves a strong genetic predisposition and transgenerational epigenome reprogramming (King and Skinner, 2020), but genetics alone is unlikely to account for such an increase: pollutants (e.g., microplastics) (Campanale et al., 2020), obesogenic diets causing increased stress on β-cells (Polsky and Ellis, 2015), infection during pregnancy (Group, 2007), sedentary lifestyle (Maja Cigrovski Berkovic et al., 2017), and microbiota shift (Knip and Siljander, 2016) also seem to be playing their parts.

**FIGURE 1:**
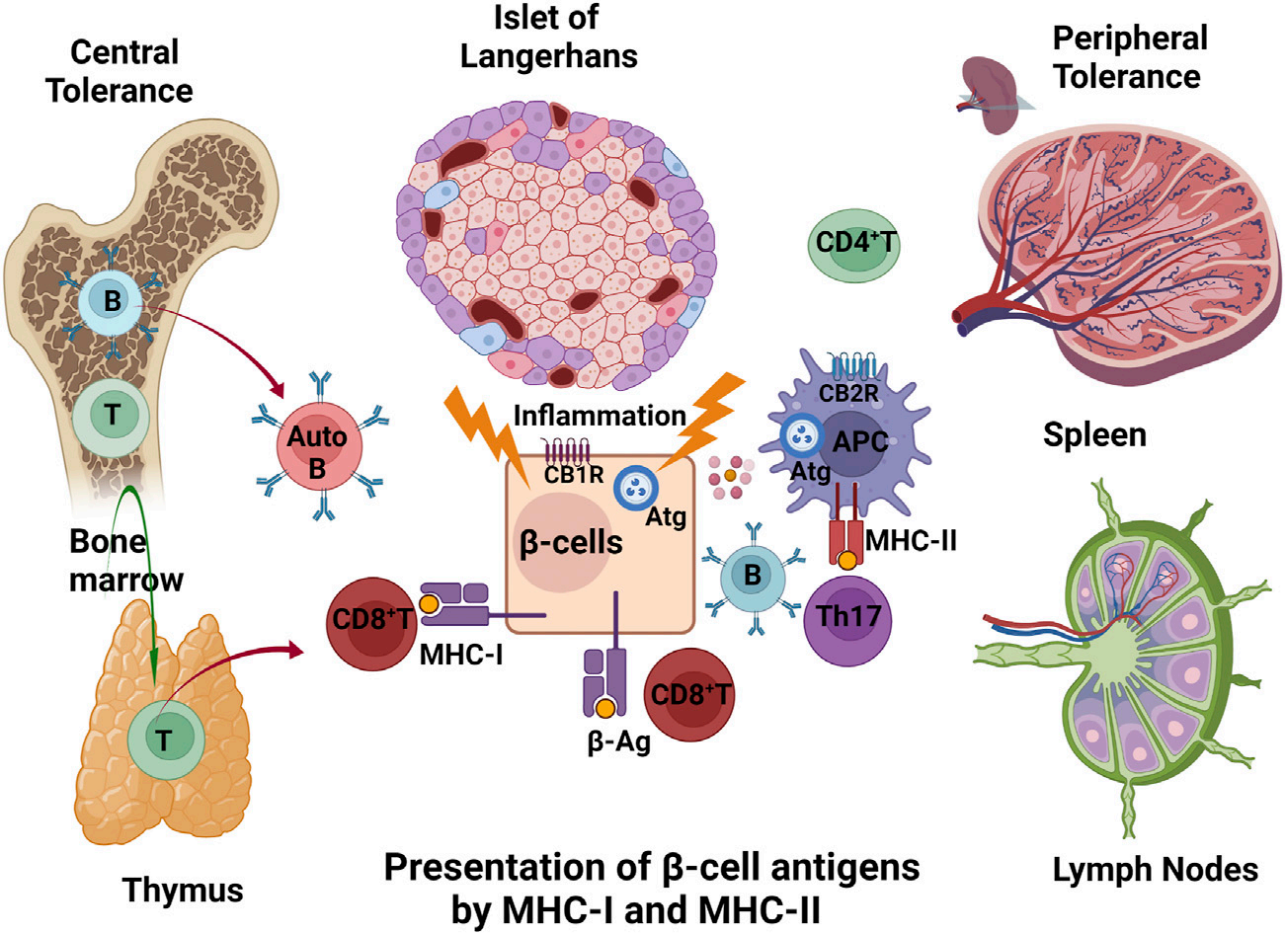
Central and peripheral breakdown of immune tolerance in T1DM: Auto-reactive cytotoxic T (CD8^+^T) and B (Auto B) cells escape from primary lymphoid (bone marrow and thymus) and secondary lymphoid organs (spleen and lymph nodes). The β-cells exposed to viral infections, an array of cytokines (IL-1β, TNF-α, IFN-γ), injury by toxins, and stress (such as increased ROS production, ER stress, post-translational modifications) conditions may present their auto/neoantigenic peptides (β-Ag) on MHC-I complexes to the cell surface, thereby attracting cytotoxic CD8^+^T cells. Autoreactive antigens that are endocytosed by antigen presenting cells (APC) activate CD4^+^Th17 T and B cells. CB1R in β-cells and CB2R in APC cells are players in the outcome to β-cells, possibly through actions on autophagy (Atg).

T1DM shows significant geographic, ethnic, age, and gender differences, with the incidence peaking between 4 and 19 years of age, then leveling off, and once again gradually increasing after the fifth decade of life (Rogers et al., 2017), indicating defective central thymic and peripheral tolerance (Littman and Rudensky, 2010; Zucchelli et al., 2005). Self-tolerance is induced in the primary lymphoid organs (thymus and bone marrow), and in spleen and lymph nodes, where self-reactive T cells are deleted, thereby guaranteeing, in normal physiology, that self-reactive T cells do not get into the circulation (Theofilopoulos et al., 2017). β-cells in islets of Langerhans exposed to viral infections (such as enteroviruses, Coxsackie B), an array of cytokines (IL-1β, TNF-α, IFN-γ), injury by toxins, and stress (such as increased ROS production, ER stress, post-translational modifications) conditions may present auto/neoantigenic peptides (β-Ag) on major histocompatibility complex molecules I (MHC-I) to the cell surface, thereby attracting cytotoxic CD8^+^T cells (Eizirik et al., 2009). CD8^+^T-lymphocytes, which recognize MHC-I peptide complexes, dominate the pro-inflammatory milieu of islet infiltration (insulitis) and are thought to be major effectors of β-cell death (Carré and Mallone, 2021). The processes in β-cell that produce MHC-I restricted antigens are poorly understood in T1DM. Autophagy (Atg) may, however, intersect with the intracellular MHC-I presentation by lessening the amount of neoantigens that are formed (Figure 1). Pancreatic β-cells are vulnerable because insulin transcription accounts for 40% of the transcriptome whereas genes involved in cellular protection such as those for chaperones, autophagy, ubiquitin, proteosome, protection from reactive oxygen species, and ER unfolded protein responses are expressed at lower levels than in other islet cell types (Benner et al., 2014; Segerstolpe et al., 2016; Diedisheim et al., 2018). Genetic susceptibility, environmental triggering, autoantibody appearance are the pre-disposing events to β-cell damage. Reduced insulin secretion and dysglycemia occur when T cells and macrophages infiltrate into the islets and gradually destroy β-cells. Finally insulin-dependent diabetes occurs when approximately 80% of the β-cells are destroyed: this is the pathological sequence of events (Eisenbarth, 1986; Insel et al., 2015; Ilonen et al., 2019). Susceptible HLA (human leukocyte antigen) DR/DQ alleles and detection of at least two autoantibodies specifically targeting β-cells are pre-diagnostic markers for T1DM (Michels et al., 2015). A humanized anti-CD3 monoclonal antibody (Teplizumab) is currently a FDA approved drug to delay occurrence of T1DM symptoms by slowing down destruction of β-cells (Herold et al., 2019). Certain natural and synthetic cannabinoids are known for their potent immunosuppressive and anti-inflammatory properties that are effective against several autoimmune diseases (Rieder et al., 2010); however, little research is carried out for early intervention with cannabinoids on T1DM risk cohorts.

### Overview of the Endocannabinoid System in relation to Islets of Langerhans

Cannabinoids are endogenously produced, lipid-derived mediators of multiple organ functions-hence the name endocannabinoids (eCBs) (Pacher et al., 2020; Lu and Mackie, 2021). The most studied eCBs are anandamide (N-arachidonoylethanolamide, AEA) and 2-arachidonoyl-sn-glycerol (2-AG), both of which are synthesized in β-cells in islets upon cellular depolarization. The whole eCB system also consists of the enzymes involved in the synthesis and degradation of the eCBs and the eCB receptors (CBRs) (Joshi and Onaivi, 2019), of which there are primarily two such receptors, CB1R and CB2R. Both are class-A G-protein-coupled receptors that function through Gi/o/q proteins and the β-arrestin signaling pathway (Aseer and Egan, 2021). In general, CB1R is highly expressed in the central nervous system while CB2R is mostly found in immune cells. However, of pertinence to this review, CBRs are also present in cells of the islets of Langerhans. There are five cell types in islets, called α-, β-, δ-, ε-, and PP-cells. These cells produce glucagon, insulin, somatostatin, ghrelin, and pancreatic polypeptide, respectively. In general, more than 50% of the islet cells are β-cells, while α-cells are the next most common cell type. Using FACS sorted mouse and human β-cells it was found that CB1R mRNA levels in mouse β-cells (GSE54973) are more than 10-fold higher than in human β-cells (GSE103383) (Benner et al., 2014; Diedisheim et al., 2018). CB1R but not CB2R mRNA was found in human β-cells by single cell sequencing also (GSE81608) (Benner et al., 2014; Xin et al., 2016). Human CB2R transcripts were found in α-, δ-, and ε-cells more than are CB1R transcripts while in PP-cells both transcripts were reported to be equally expressed (Xin et al., 2016). Low basal expression of CNS-enriched CB1R is also present in myocytes, adipocytes and hepatocytes (González-Mariscal et al., 2016), while leukocyte-enriched CB2R is found in adipocytes, neurons and microglia (Karaliota et al., 2009; Liu et al., 2020a).

Exogenous cannabinoids are also available in marijuana plants; ∆9-tetrahydrocannabinol (∆9–THC), cannabidiol (CBD), and (−)-β-caryophyllene (BCP). ∆9–THC is a ligand for both CB1R and CB2R, while BCP is a selective CB2R ligand. All the molecular target receptors of CBD are unknown but CBD is thought to be involved in enhancing serotonin 5-HT_1A_ receptor and transient receptor potential cation channel (TRPV1) activity (Pacher et al., 2020; Lu and Mackie, 2021). Inverse agonists (antagonism) of CB1R were developed 20 years ago as treatments for obesity: however, rimonabant that did come into use for that purpose was quickly withdrawn because of severe adverse psychiatric effects (Sam et al., 2011). Further development of CB1R antagonists and inverse agonists by pharmaceutical companies was then halted. Unrelated to CNS effects, we have shown that, in regards to β-cells, peripheral inhibition of CB1R leads to: improved insulin secretion in response to glucose; enhanced responses to incretins; increased intracellular cAMP levels; resistance to inflammation from high fat diets; and protects against apoptosis due to toxins and high fat diets (Gonzalez-Mariscal et al., 2018). CB2R has a yin-yang relationship with CB1R structurally and functionally (Shao et al., 2016; Li et al., 2019) in the context of cell types. While activation of CB2R has general anti-inflammation effects (Basu and Dittel, 2011; Wu et al., 2018), cell type specific CB1R deletion in β-cells, myocytes, and hepatocytes has anti-inflammatory effects in mice (Gonzalez-Mariscal et al., 2018; González-Mariscal et al., 2019; Kim et al., 2020). CB2R enriched in various cell types of the immune system (Fernández-Ruiz et al., 2007; Hu et al., 2020) appears to result in little or no adverse CNS effects, unlike CB1R, when activated (Buckley et al., 2000; Turcotte et al., 2016). CB2R activation in the immune system is also thought to be anti-inflammatory and pro-tolerance and therefore may aid in preventing autoimmune-mediated self-destruction (Eisenstein and Meissler, 2015). There are rich sources of natural and synthetic CB2R selective agonists that potentially could be investigated for intervention at the pre-symptomatic phase of T1DM. Hemp seeds (Pellati et al., 2018), cloves (Siani et al., 2013), black pepper (Geddo et al., 2019), and manacá (Galdino et al., 2012) with high content of β-caryophyllene are widely consumed in India, China, and Brazil (Patterson et al., 2019). A synthetic cannabidiol quinone derivative (THP-101), a CBD analog with CB2R agonist properties, added another potential remedy for autoimmune diseases (Navarrete et al., 2018). We will now analyze the literature with regards to the possible molecular mechanisms whereby regulating activity of CB2R might have therapeutic potential in the spectrum of T1DM with emphasis on molecular evolution, immune tolerance, anti-inflammation, autophagy, and extracellular vesicles secretion.

### CB2R and Evolution

**FIGURE 2:**
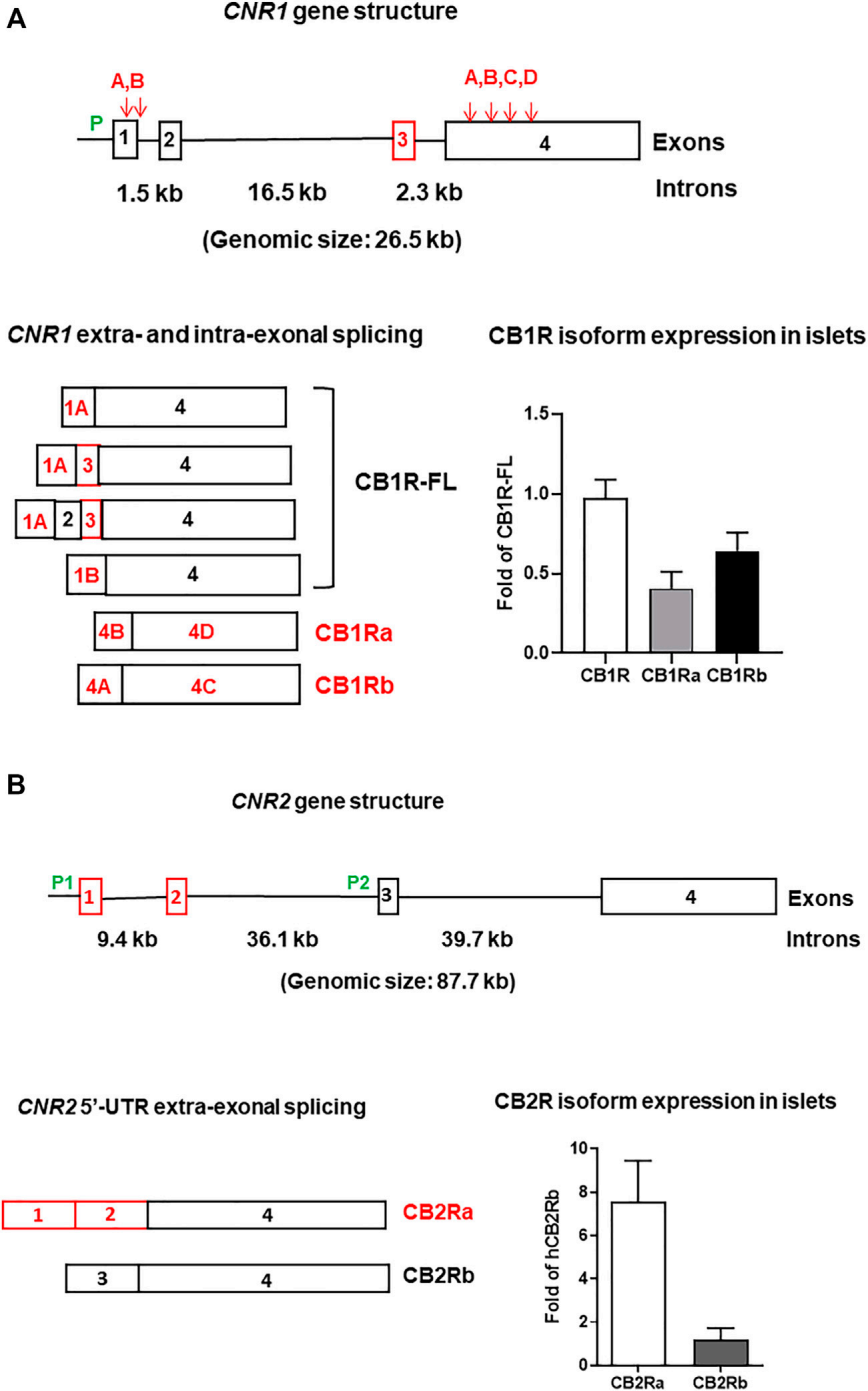
Human *CNR1* **(A)** and *CNR2* **(B)** gene structures, their alternatively spliced isoforms, and expression in pancreatic islets. P (green lettering) represents promoters. Exons are open boxes and introns solid lines. The exon numbers are inside the open boxes and intron sizes are marked in kb (kilobase). Downward arrows and capital letters are at the intra-exonal splicing sites. Red letterings, boxes, and arrows represent human specific isoforms, exons, and splicing sites, respectively. The reference of CB1R isoform islet expression (n = 6) is full-length CB1R (CB1R-FL) and the reference of CB2R isoform islet expression (n = 3) is CB2Rb (unpublished data from Diabetes Section, LCI/NIA/NIH).

No *CNR1*/*CNR2* orthologs are present in protostome invertebrates even though specific enzymes necessary for eCB synthesis and breakdown are present, as are vanilloid-type ion channels that could serve as eCB receptors (Elphick, 2012). A single *CNR1*/*CNR2* ortholog is present in genomes of deuterostome chordates such as the sea squirt *Ciona intestinalis* (ciCBR, 423 AA) and lancelet *Branchiostoma floridae* (bfCBR, 410 AA), expressed in branchial pharynx, heart, cerebral ganglion, testis, ovaries, and gut (Elphick, 2007; Elphick et al., 2003; Matias et al., 2005). The primitive chordate *CNR* gene has only one promoter without upstream exons encoding different 5′UTRs (McPartland et al., 2006) as is observed in *CNR1* and *CNR2* of mammalian species (Zhang et al., 2015; Liu et al., 2019). Human CB1R (472 AA) is enriched in neurons and is more homologous to chordate ciCBR and bfCBR (Elphick, 2007). CB2R (360 AA) is enriched in the immune system (Liu et al., 2020a) and *CNR2* likely arose due to vertebrate genome duplication about 500 million years ago (Elphick, 2002) when the adaptive immune system and major histocompatibility complex class I (MHC-I) and class II (MHC-II) are reported to have first appeared in jawed fish (Flajnik and Kasahara, 2010; Wu et al., 2021). During mammalian evolution, human gene exonization (Li et al., 2018) and splicing isoform evolution (Zhang et al., 2017a) contributed to multiple upstream exons with a single promoter in *CNR1* and two promoters in *CNR2* (González-Mariscal et al., 2016; Liu et al., 2009) to diversify eCB signaling in a specific cell type context (Marti-Solano et al., 2020) and the genomic size of *CNR2* is more than 3-fold larger than that of *CNR1* (Figures 2A,B). Human *CNR1* has one promoter and four exons that are spliced into six variants including two human-specific N-terminal amino acid (AA) altered isoforms (González-Mariscal et al., 2016), while *CNR2* gene has two separate promoters and four exons that are spliced into CBR2a (human-specific) and CBR2b isoforms, encoding the same peptide sequences (Liu et al., 2019). *CNR1* contains human-specific exon-3 and intra-exonal splice sites of exon-1 and coding exon-4, creating altered N-terminal AA isoforms of CB1Ra and CB1Rb (González-Mariscal et al., 2016). *CNR2* contains human-specific exon-1 and -2 encoding isoform CB2Ra that is under control of human-specific promoter-1, whereas the promoter-2 controls expression of generic exon-3 and -4 encoding CB2Rb isoform that is preferentially expressed in immune system (Liu et al., 2009). The human-specific evolution of eCB system could explain that THC is rewarding to humans but not rodents (Zhang et al., 2015; Han et al., 2017). Although CB1R is predominantly expressed in mammalian brain, we observed low basal expression of CB1R in many peripheral tissues, and interestingly the liver of humans has a predominant N-terminal intra-exonal spliced isoform (CB1Rb), expression of which is increased by obesity (González-Mariscal et al., 2016). Global CB1R knockout mice, though fertile, have detrimental phenotypes of increased morbidity and weight loss, agitation, and early death (Zimmer et al., 1999). In contrast, global CB2R knockout mice, while also fertile, appear healthy unless challenged with endotoxins (Kapellos et al., 2017) and high fat-sugar diet (Agudo et al., 2010), implying that manipulation of CB2R might not have severe adverse CNS side effects.

### CB2R and Immunity

As stated above, CB2R is predominantly expressed in the immune system with a rank order of B-cells (B-lymphocytes) > granulocytes > dendritic cells > macrophages > CD8^+^Tcells > natural killer T-cells > CD4^+^T-cells > natural killer cells (Galiègue et al., 1995; López et al., 2018). CB2R expression is highly inducible during inflammatory processes and its activation polarizes macrophages from a classical pro-inflammatory (M1) state to an alternative anti-inflammatory (M2) state (Braun et al., 2018). For example, there is a 40-fold increase in CB2R expression by the 5th day in mouse right brain cortex when the right middle cerebral artery is occluded for 30 min and causes right cortical ischemia. This gradually subsides to the basal level by the 10th day to levels similar to those of the left non-ischemic cortex (Yu et al., 2015). Activation of CB2R by GP1a (a CB2R agonist) reduced HLA DQ expression by 10-fold in an ipsilateral mouse brain hemisphere that was stereotactically injected with HIV-1 infected human monocyte-derived-macrophages in comparison with the non-injected contralateral hemisphere (Gorantla et al., 2010). Both CB2Ra and CB2Rb isoforms are activated by inflammation and psychiatric stress (Zhang et al., 2015). Activation of CB2R resulted in decreases in cell surface expression of MHC-II molecules and the pro-inflammatory cytokines IL-1β and IL-12p40 (Mestre et al., 2005). Although CB2R is enriched in the immune system, we observed CB2R expression in microglia, as might be expected, and neurons in different mouse brain regions (Liu et al., 2020a). Interestingly, we found that CB2Ra but not CB2Rb is expressed in human testis (Liu et al., 2009) and Nielson et al. reported that CB2Ra is involved in germ cell maturation and is localized in the cytoplasm of late spermatocytes and round spermatids but not early spermatocytes (Nielsen et al., 2019). We found that the CB2Ra transcript levels are about 8-fold higher than that of CB2Rb in human islets (Figure 2B), indicating that the upstream promoter is more active in cell types outside of immune system (Zhang et al., 2017b). The expression of CB2R in non-immune system implies that CB2R is not only involved in MHC class II (MHC-II) immune cell response (Gorantla et al., 2010) but also in pan MHC class I (MHC-I) cells that present oncogenic and invading intracellular virus antigens to cell surface (Karmaus et al., 2013). Whether CB2R plays a role in immune tolerance in T1DM is currently not reported.

### CB2R and Autoimmune Diseases

**FIGURE 3:**
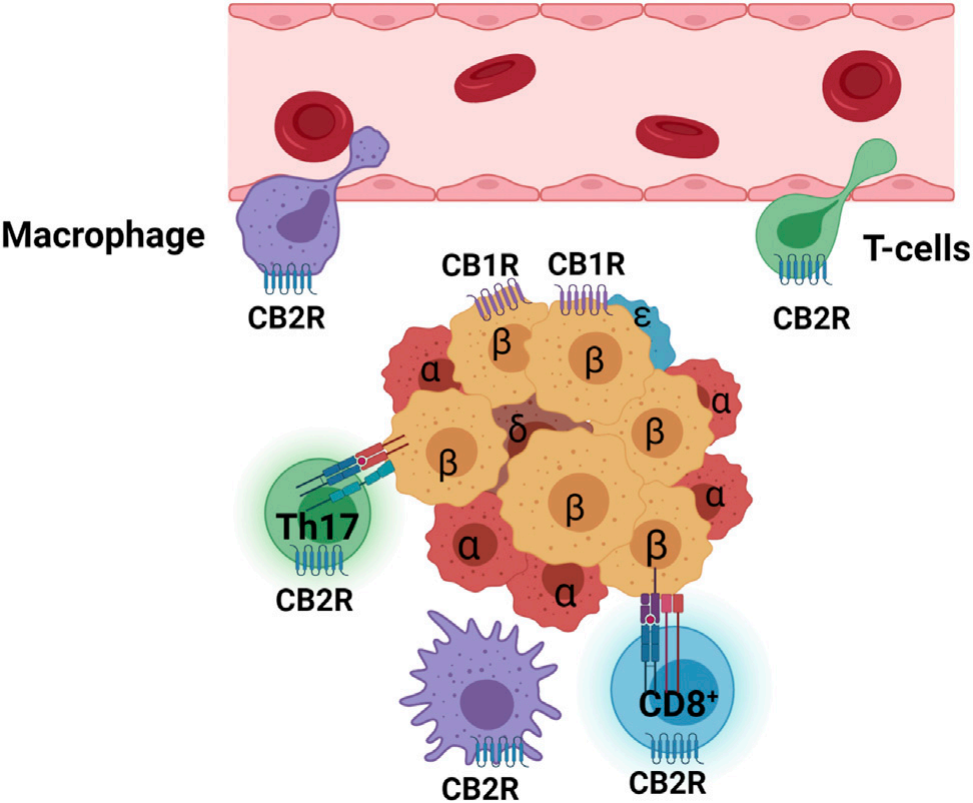
Activated CB2R suppresses macrophage (purple) and T cell (green) infiltration from endothelium barrier of blood vessel and inhibits M1 macrophages (purple ameba shape), self-reactive HMC-I CD8^+^T cells (blue with halo), and CD4^+^Th17 cells (green with halo). Islet cell types are marked with Greek letters (α, β, δ, ε). Purple serpentine represents CB1R in β-cells and blue serpentine CB2R in immune cells.

T1DM shares genetic and phenotypic comorbidity with other autoimmune diseases and CB2R activation can ameliorate symptoms of multiple sclerosis (Annunziata et al., 2017), thyroiditis autoimmune diseases (Alcigir et al., 2017), celiac disease (Tortora et al., 2020), Crohn’s disease (Leinwand et al., 2017), and rheumatoid arthritis (Gui et al., 2014). Several human leukocyte antigen (HLA) gene polymorphisms of MHC class I and II (Noble and Valdes, 2011), insulin gene short VNTR (variable number tandem repeat) (Bennett et al., 1995), and a nonsynonymous *CNR2* SNP (Q63R) are risk alleles that co-segregate with several autoimmune diseases (Rossi et al., 2012; Mahmoud Gouda and Mohamed Kamel, 2013; Bellini et al., 2015; Ismail and Khawaja, 2018; Strisciuglio et al., 2018). However, we could not find any study of CB2R and its level of activation in T1DM in the literature. Damage to β-cells in T1DM patients is initiated by recruiting circulating T cells and macrophages that migrate across vascular endothelium cells by orchestrated multimodal movements; tethering, rolling, arrest, firm adhesion, and migration that are mediated by selectins, integrins, and cytoskeleton molecules (Alon and Shulman, 2011). Activation of CB2R has been shown to reduce formation of leukocyte lamellipodia by downregulation of integrins (ITGA4 and ITGB2) and small GTPases (RAC1 and RHOA) that promote adhesion and cytoskeleton dynamics, respectively, necessary for trans-endothelium migration (Rom et al., 2013). Activation of CB2R has also been shown to protect from tissue damage by controlling recruitment of CD34^+^ myeloid progenitor cells and neutrophils, reducing infiltration CD4^+^ T-lymphocyte subset of T helper 17 (Th17) cells (Cencioni et al., 2010), suppressing CD8^+^T lymphocytes (Joseph et al., 2004), and regulating macrophage function by altering expression of pro- and anti-inflammatory cytokines and their receptors (Palazuelos et al., 2008; Kapellos et al., 2017; Kapellos et al., 2019) (Figure 3).

### CB2R and Immune Tolerance

**FIGURE 4:**
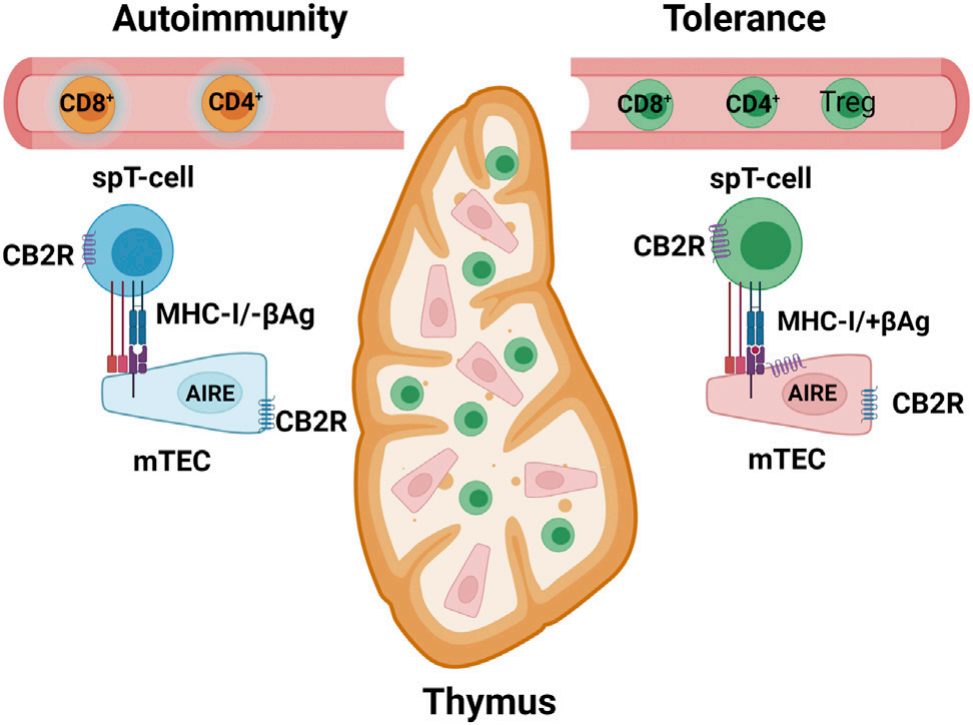
MHC-I β-cell restricted antigen (+βAg) or no antigen (−βAg) presentation in medullary epithelium cells (mTEC) inside thymus. AIRE, autoimmune regulator for ectopic expression of β-cell-specific genes. spT cells, single-positive CD8^+^ or CD4^+^ T cells. Yellow represents self-reactive and green self-tolerance T cells. Purple serpentine represents CB1R in β-cells and blue serpentine CB2R in immune cells.

The intracellular autoantigens of β-cells are processed by ubiquitin–proteasome system into small peptides of 8–11 AAs that are transported into the endoplasmic reticulum (ER) by TAP (transporter associated with antigen processing), and then bind to MHC-I with assistance of chaperone, and further translocated to plasma membrane *via* Golgi apparatus (Strehl et al., 2005). Cytotoxic CD8^+^T cells with specific T cell receptors (TCRs) for the autogenic peptides are activated and exert apoptotic effects on β-cells (Gupta et al., 2006). CB2R is expressed in thymus (Schatz et al., 1997) where autoimmune regulator AIRE (mutated in APS-1, autoimmune polyendocrine syndrome type 1) stimulates ectopic expression of intracellular T1DM autoantigens (e.g., insulin and GAD65) in medullary thymus epithelium cells (mTECs) in which the endogenous peptides are presented to the cell surface by MHC-I (Alexandropoulos et al., 2015). Insulin gene (*IN*S) with long-VNTR alleles promotes higher expression of insulin in mTECs that present more insulin peptides to educate CD8^+^T cells not to be self-reactive (Fan et al., 2009; Mathis and Benoist, 2009; Levi and Polychronakos, 2013). Proteasome processing of T1DM autoantigens for MHC-I presentation requires unfolding of protein monomers and is not capable of unfolding oligomeric insulin. In that case, autophagosomes and lysosomes are involved in the autoantigen presentation in mTECs (Yedidi et al., 2017; Øynebråten, 2020). CB2R expression is 6-fold higher than CB1R in mTECs (GSE89892) within the thymus (Guha et al., 2017) and is upregulated in activated T cells where very little or no CB1R is found (Schatz et al., 1997; Coopman et al., 2007), and therefore the pro-autophagy function of CB2R may prevent insulin from being mispresented. Clonal selection of immunosuppressive regulatory T cells (CD4^+^FOXP3^+^Treg cells) prevents self-reactivating T cells from exiting into the circulation (Kraj and Ignatowicz, 2018). Deletion of FOXP3^+^Treg accelerates onset of T1DM (Mariño et al., 2009) and infusion of FOXP3^+^Treg cells delays the onset of T1DM in young NOD mice (Spence et al., 2018). CB2R expression is preferentially induced in FOXP3^+^Treg-cells and the agonist GP1a enhances FOXP3^+^Treg immunosuppressive function in Crohn’s disease (Leinwand et al., 2017). Pancreatic β-cells do not express MHC-II because it is restricted to professional antigen presenting cells (APCs), such as CB2R enriched macrophages, dendritic and B cells (Roche and Furuta, 2015). The β-cells secrete and present the autoantigens that are endocytosed by APCs and fused with lysosomes, and further processed by endosome-lysosome pathway (Lundberg and McDevitt, 1992) to peptides of 12–25 AAs (Wu et al., 2021) that bind to MHC-II-Ii (Invariant chain) complex in ER and translocate *via* Golgi apparatus to endolysosomes in which Ii is cleaved by cathepsin L and the remaining CLIP (class II-associated invariant chain peptide) prevents autoantigen presentation to APCs that regulate CD4^+^T cell differentiation (Jurewicz and Stern, 2019). During inflammation, the activated APCs present more β-cell-derived neo- and autoantigen peptides that stimulate pathological Th17 cytotoxic cell expansion in lymph nodes and in circulation (Honkanen et al., 2010). Peripherally, in the secondary lymphoid organs (lymph nodes, spleen, tonsils, and mucous membranes), pathologic autoreactive CD4^+^T helper cells (Th17/Th1^+^) cause breakdown of peripheral tolerance and inflammation (Cencioni et al., 2010; Fava et al., 2016). The CB2R synthetic agonist JWH015 reduces IL-17, TNF-α, and IFN-γ secreted by Th17 cells, and suppresses anti-CD3/anti-CD28 induced CD4^+^ and CD8^+^T cell proliferation by reducing T cell growth factor IL-2 (Cencioni et al., 2010) (Figure 4). Whether CB2R is involved in breakdown of central and peripheral immune tolerance in T1DM is unknown.

### CB2R and Inflammation

**FIGURE 5:**
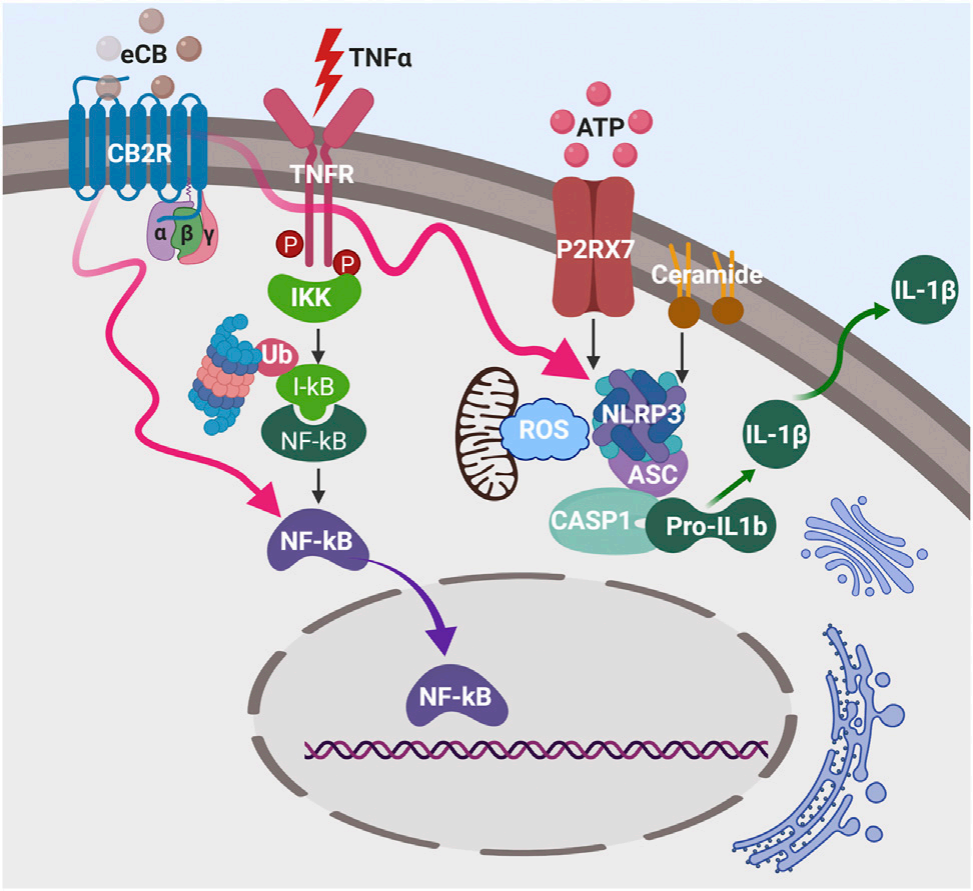
Activation of plasma membrane CB2R pathway inhibits NLRP3 inflammasome complex and NFκB activation (red wavy arrows) during inflammation that is initiated by TNFα, ATP, and ceramide, thereby reduces IL-1β secretion.

Inflammatory components of the innate immune system such as toll-like receptors (TLRs), NLRP3 (NLR family pyrin domain containing 3) inflammasome, and IL-1β contribute to the etiology of T1DM and their activation recruits inflammatory T cells and macrophages into islets where they are cytotoxic to β-cells (Grishman et al., 2012). CB2R is prominently upregulated by inflammation and a selective synthetic CB2R agonist, JWH-133, inhibits the TLR4/NF-κB signaling pathway, reduces infiltration of immune cells across endothelium, thereby mitigating against immune-mediated tissue damage (Yu et al., 2015; Chen et al., 2019; Jing et al., 2020). Another selective synthetic CB2R agonist, HU-308, inhibits NLRP3 inflammasome expression and activation, leading to reduction of IL-1β secretion from macrophages and microglia in a mouse model of dextran sulphate sodium (DSS)-induced colitis and experimental autoimmune encephalomyelitis (EAE) (Shao et al., 2014; Ke et al., 2016). The naturally occurring CB2R selective agonist, β-caryophyllene, inhibits hypoxia-induced cytotoxicity by decreasing proinflammatory cytokine secretion of IL-1β, TNF-α, and IL-6 in a murine microglia cell line, BV2 (Guo et al., 2014). A selective CB2R agonist, AM124, used in a rat model of complete Freund’s adjuvant (CFA)-induced inflammatory dermatitis decreases the expression of IL-1β, IL-6 and TNF-α (Nascimento et al., 2012; Su et al., 2012). It has been shown that, under stress damaged mitochondria release mtDNA into the cytosol and enhance production of reactive oxygen species (ROS) in an inflammasome-dependent manner in both macrophages and Th17^+^T cells (Nakahira et al., 2011; Kaufmann et al., 2019). Since the inflammasome is exquisitely sensitive to nucleic acid and ROS, the activated inflammasome produces the proinflammatory cytokines, IL-1β and IL-18, resulting in vicious inflammatory cycle (Nakahira et al., 2011). Since CB2R agonists stimulate calcium release from lysosomes that tether and transfer calcium to mitochondria to reduce NLRP3 inflammasome activation (Peng et al., 2020) and ROS production they may be possible therapeutic agents to mitigate inflammation induction (Figure 5). In sum, although CB2R activation reduces the proinflammatory cytokines in certain disease models, there is no published research on the possibility of CB2R activation being protective of β-cell destruction due to proinflammatory cytokine-induced cytotoxicity during onset of T1DM.

### CB2R and Autophagy

Malfunction of intracellular membrane trafficking is involved in autoantigen presentations by MHC-I and -II and autophagy compensates compromised protease activities of ubiquitin-proteasome system in antigen presentation and impaired autophagy has been documented in T1DM (Valecka et al., 2018; Muralidharan et al., 2021). Autophagy is classified as macro-, micro-autophagy, and chaperone-mediated autophagy that share intracellular proteolytic pathway and membrane trafficking machinery as MHC antigen presentation pathways and are potentially able to cross-present β-cell-derived autoantigens (Valecka et al., 2018; Germic et al., 2019). Autophagy is an evolutionarily conserved mechanism that helps all cells degrade and recycle biological materials under a range of situations, including ER stress. Specifically, macroautophagy (hereafter referred to as autophagy) involves the transport of cargo contained in double-membraned autophagosomes to the lysosome (Parzych and Klionsky, 2014). Hyperglycemia and the buildup of ROS, as well as endoplasmic reticulum (ER) stress, are known to disturb β-cell homeostasis (Gerber and Rutter, 2017; Newsholme et al., 2019). Furthermore, excessive ROS can damage proteins and organelles, making it more difficult for the cell to activate its adaptive stress response systems. Endogenous activities that help to pacify these cellular stressors and restore homeostasis are thus crucial for β-cell survival. In this context, the role of autophagy in maintaining β-cell homeostasis and increasing cell survival has been examined (Marasco and Linnemann, 2018; Vivot et al., 2020).

CB2R, as described above, is mainly located in the cells of the immune system and participates in the modulation of immune responses (Basu and Dittel, 2011). Moreover, CB2R stimulation has been shown to promote autophagy in various cellular and animal models. Notably, JWH-133 reduced the expression of lipopolysaccharide (LPS)-induced inflammatory genes in autophagy related protein 5 (ATG5)-sufficient macrophages but not in ATG5-deficient cells, and JWH-133 treatment also protected mice from alcohol-induced liver inflammation and steatosis but was not protective in mice lacking ATG5 in myeloid cells (Denaës et al., 2016). As a result, activation of CB2R in macrophages protects against alcohol-induced steatosis through an autophagy-dependent route (Denaës et al., 2016). Selective activation of CB2R with HU308 had a cardio-protective effect against diabetic cardiomyopathy and protected the cardiomyocytes by promoting autophagy *via* the AMPK-mTOR-p70S6K signaling pathway when maintained under the stress of high glucose (Wu et al., 2018). In addition, autophagy induction and p62-mediated Nrf2 deactivation are linked to CB2R activation-induced osteoblastic differentiation *in vitro* (Xu et al., 2020). The synthetic CB2R agonist AM1241 protects rats from cardiac ischemia-reperfusion injury by triggering autophagy through activation of the Pink1/Parkin pathway (Liu et al., 2021). HU-308 (a CB2R agonist) promotes autophagy, inhibits the NLRP3 inflammasome, and protects mice from autoimmune encephalomyelitis (Shao et al., 2014). JWH133 orchestrates neuronal autophagy in the hippocampus of developing rats with status epilepticus through modulating the mTOR signaling pathway (Wu et al., 2020a). Taken together, these studies imply that activating the CB2R promotes autophagy *in vitro* and *in vivo*. It is therefore reasonable to propose that CB2R plays a critical role in autophagy processes and consequently may protect from the autoimmunity of T1DM by this mechanism.

Antigen-presenting cells such as dendritic cells (DCs) that lack the core autophagy machinery that enables ATG8 (autophagy related protein 8) lipidation, for example, have increased surface MHC-I expression, which is linked to hyper-reactive CD8^+^T cell responses (Hubbard-Lucey et al., 2014). Blocked internalization and degradation of MHC-I molecules, which involves recruitment of MHC-I molecules *via* (probably membrane coupled) LC3B (microtubule-associated proteins 1A/1B light chain 3B), are among the mechanisms underlying loss of components in the autophagy machinery in MHC-I restricted antigen presentation in DCs (Loi et al., 2016). Interestingly, CB2R has been reported to regulate autophagy in non-pancreatic cells. Nevertheless, there have been no investigations on CB2R-mediated autophagy in pancreatic islets or β-cells, as there have been for antigen processing, immune cell differentiation, and macrophage migration in the context of airway immunomodulation (Carayon et al., 1998; McCoy et al., 1999). Because CB2R activation is known to increase autophagy in other cellular/tissue contexts, CB2R agonists could be a viable treatment option to control CD8^+^T cell response and MHC-I antigen presentation leading to stress conditions in pancreatic islets during T1DM initiation and progression. Hence, future research into the novel role of CB2R in T1DM and its complications, particularly in pancreatic islets and its immune cell infiltration, would be worthwhile.

**FIGURE 6:**
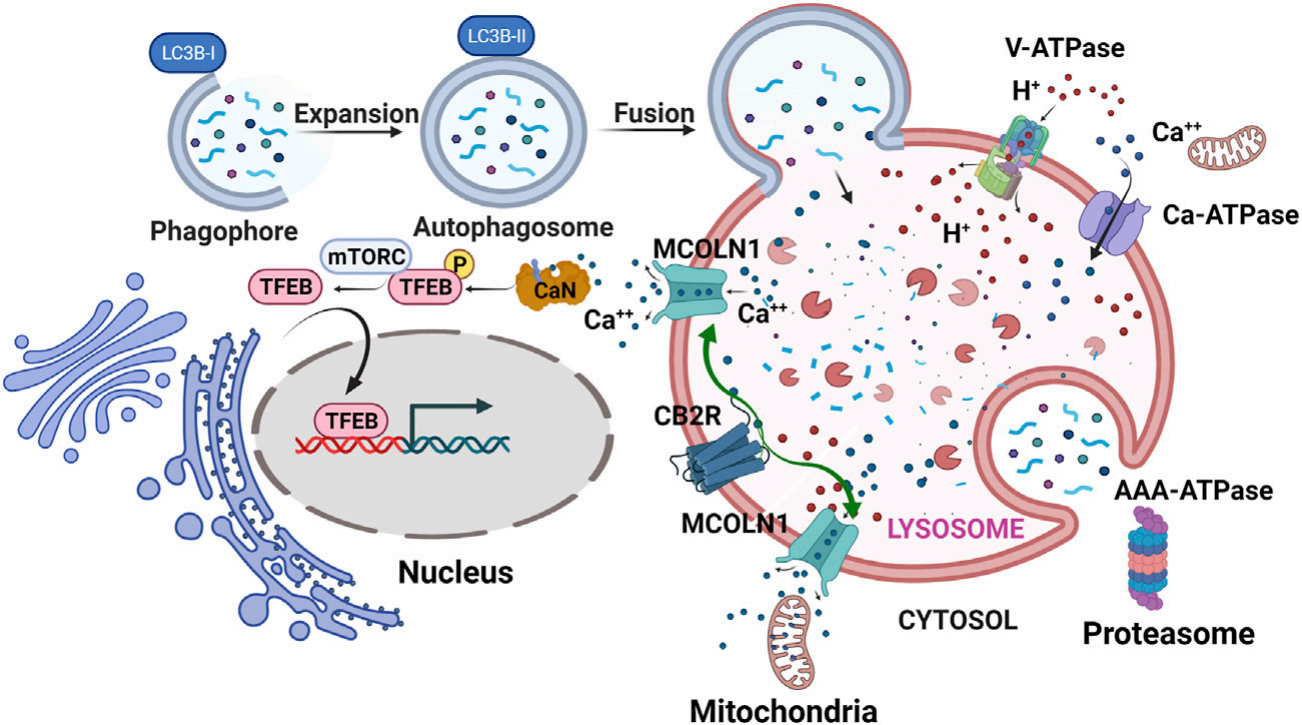
CB2R pro-autophagy effect. Lysosomal CB2R activation results in release of intra-lysosomal Ca^2+^ through MCOLN1 (Mucolipin TRP Cation Channel 1). The released Ca^2+^ tethers mitochondria to lysosomes and some Ca^2+^ ions are transferred to the mitochondria where less ROS is then produced. Calcineurin (CaN) is also activated that then dephosphorylates TFEB causing its translocation to nucleus, downstream of which autophagosomes and lysosomes are generated. Green arrows represent CB2R stimulation of MCOLN1 to release Ca^2+^ ions that enter mitochondria and activate calcineurin (CaN) for TFEB nuclear translocation. V-ATPase, vacuolar-type ATPase; Ca-ATPase, Calcium ATPase; AAA-ATPase, ATPases Associated with diverse cellular activities.

CB1R might regulate MHC-I in β-cells and CB2R regulate MHC-II in immune cells since CB1R and not CB2R is found in β-cells (Benner et al., 2014). Autophagy is highly dynamic, ATP-dependent, and maintains photostatic homeostasis in β-cell when proteosome machinery is compromised and could not properly present antigenic peptide through MHC-I in β-cell (Broca et al., 2014). Targeting autophagy pathways regulated by cannabinoids for prevention of T1DM is a pathway worth investigating as a way to prevent presentation of auto- and neo-antigens to APCs (Fierabracci, 2014). Intracellular CB1R and CB2R also play important roles in metabolism and immunity (Brailoiu et al., 2014; Brailoiu et al., 2011). Activation of mitochondria CB1R dysregulates astrocyte glucose metabolism and promotes glycolysis in activated T cells (Jimenez-Blasco et al., 2020). The activation also modulates inflammation by reducing microglia oxygen consumption (Beji et al., 2020) and reduces mitophagy (Kataoka et al., 2020). Rimonabant was found to protect liver ischemia-induced inflammation through increasing autophagic flux, as illustrated by upregulation of proteins in the autophagy pathway, p62 (SQSTM1), Beclin-1 and LC3B-I to LC3B-II conversion (Rezq et al., 2021). On the other hand, CB2R is localized intracellularly at endolysosomes and microinjection of 2-AG into bone sarcoma U2OS cells induced faster and higher amplitude Ca^2+^ release from intracellular calcium pools (Brailoiu et al., 2014) than cytoplasmic CB2R activation. Calcineurin is then activated by calcium and dephosphorylates transcription factor EB (TFEB opposing mTORC1 kinase) (Medina et al., 2015). Dephosphorylation of autophagy Top-Chef TFEB (Cuervo, 2011; Settembre et al., 2011) causes its activation and translocation to the nucleus. The nuclear TFEB subsequently promotes lysosome biogenesis and exocytosis, and upregulates genes involved in autophagy (Settembre et al., 2011), implying links between CB2R and downstream effects on enhancing autophagy. Indeed, increased expression of CB2R is associated with enhanced autophagic flux as shown by enhanced LC3B-I to LC3B-II conversion, upregulation of Beclin-1, and increased p62 degradation in hFOB 1.19 cells derived from osteoblasts (Xu et al., 2020). Furthermore, mice treated with HU308 had some protection from diabetic cardiomyopathy and reduced ischemic myocardial infarction size through similar increases in autophagic flux (Wu et al., 2018; Xu et al., 2020). We propose that CB2R activation causes Ca^2+^ release from endolysosomes through the lysosomal calcium efflux channel MCOLN1 (transient receptor potential mucolipin 1) that not only causes dephosphorylation of TFEB and results in its nuclear translocation, but also increases lysosome contact sites with mitochondria and aids in actively transfer of Ca^2+^ into mitochondria, resulting in reducing their production of ROS, and increasing energy supply for lysosome biogenesis (Peng et al., 2020). Calcium influx and efflux regulate immune cell activation that is intertwined with autophagy (Jia et al., 2013). CB2R’s influence in autophagy may be that it participates in the delicate intracellular calcium homeostasis that regulate neo- and auto-antigen presentation in APC cells (Figure 6). The yin-yang relationship of CB1R and CB2R actions in islets illustrates the potential therapeutic of Δ^9^-tetrahydrocannabivarin (THCV), a dual antagonist/agonist for CB1R and CB2R respectively, for treating T1DM that may improve pancreatic β-cell function (Abioye et al., 2020), possibly by promoting autophagy through antagonism of CB1R within β-cells and agonism of CB2R in APC cells (Jadoon et al., 2016).

### CB2R and Obesity

**FIGURE 7:**
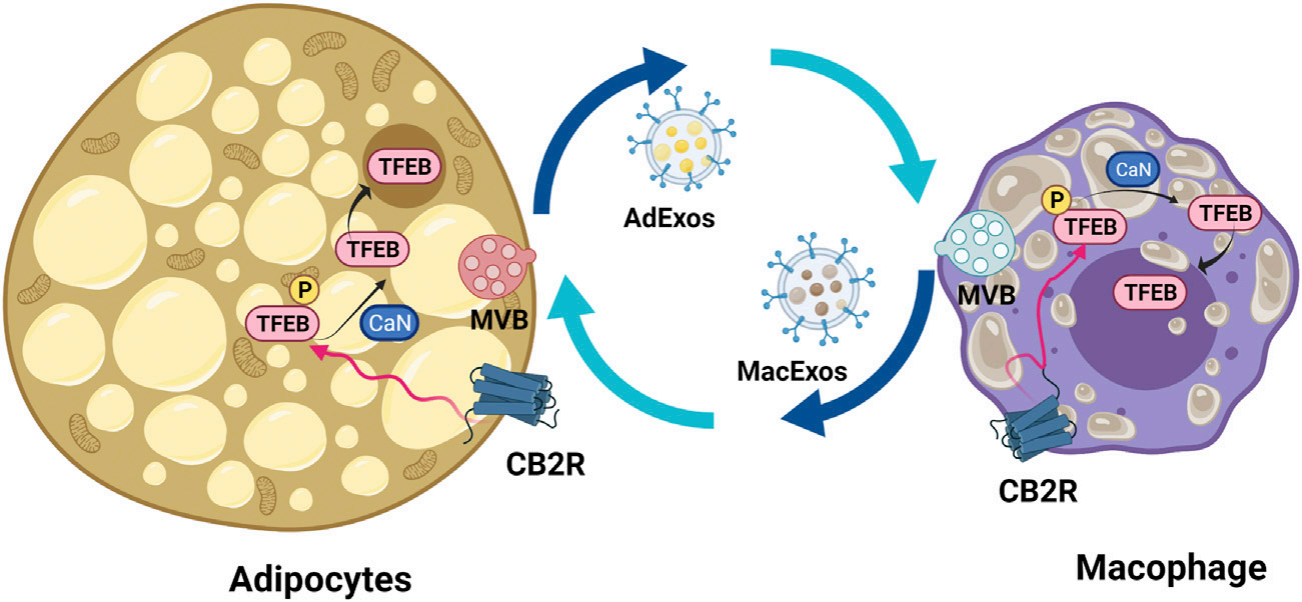
Lipid droplet-loaded extracellular vesicle recycling between adipocyte and adipose tissue macrophage. Activation of CB2R results in TFEB translocation to nucleus and activation of autophagy and lysosome biogenesis, thereby improving the lipid cycle between adipocytes and macrophages. ApExos represents adipocyte exosomes and MacExos macrophage exosomes. The adipocyte multivesicular body (MVB) is represented with pink color and macrophage light blue color.

Obesity increases risk for T1DM, especially in children (Polsky and Ellis, 2015). Adipose tissues from obese individuals contain enlarged adipocytes that secrete inflammatory cytokines such as IL-6, soluble IL6R, TNF-α and MCP-1 into circulation and thereby induce infiltration of macrophages (Lauterbach and Wunderlich, 2017). Obesity associated chronic inflammation causes insulin resistance in muscle, liver, heart, and the endothelial layer of blood vessels by stimulation of Ser/Thr phosphorylation of IRS1 proteins, and by inhibiting insulin receptor signaling both directly and indirectly through action of JNK and IKK-β (Chia and Egan, 2020). eCBs are components of the paracrine and endocrine pathways that regulate appetite/satiety and fatty acid metabolism through central and peripheral actions (Lynes et al., 2019; Behl et al., 2021). Low levels of CBRs are present in mature adipocytes and in primary cultures of rat adipocytes, and under obese conditions, their CB1R expression increases while CB2R decreases (Karaliota et al., 2009). Functions of dually and singly expressed CB1R and CB2R depends on the cell context and the low basal expression of CB1R in peripheral tissues and CB2R in brain regions exert cell type specific amplifiable actions similar to Pascal’s leverage (Kim et al., 2020; Liu et al., 2017; Xi et al., 2011), e.g., activation of CB2R induces hyperpolarization of hippocampal and cortical neurons (Stempel et al., 2016; Stumpf et al., 2018). The dynamic ranges of CB1R and CB2R mRNA levels from CNS to peripheral tissues are among the highest of the GPCR superfamily (Liu et al., 2020a) and CB2R is more inducible than is CB1R in the setting of obesity-related inflammation (Yu et al., 2015; Wu et al., 2020b). Pharmacological and genetic inhibition of total-body CB1R results in significant weight loss (Sam et al., 2011; Zimmer et al., 1999). We also found that ablation of CB1R in β-cells, myocytes, and hepatocytes lessens inflammation and improves metabolism in those tissues, especially when animals are placed on high-fat, high-sugar diets (Gonzalez-Mariscal et al., 2018; González-Mariscal et al., 2019; Kim et al., 2020). Peripherally restricted CB1R inverse agonists (Cinar et al., 2020) and CB1R blocking antibodies show promising anti-obesity effects and are under early-stage clinical development (Dao and François, 2021). On the other hand, CB2R germline knockout mice are reported to have increased food intake and total body fat content, especially as they age (Agudo et al., 2010; Alshaarawy et al., 2019). Activation of CB2R promotes β-oxidation (Zheng et al., 2013) and reduces body fat in diet-induced obesity by inhibiting pro-inflammatory M1 macrophage polarization and inducing M2 macrophages to secrete anti-inflammatory cytokines (Wu et al., 2020b). Recently LC3B dependent extracellular vesicle (EV) loading/secretion (LDELS) of lipid droplets was found to be dependent on LC3B-II conjugation to lysosomes, lipidation by ATG7 (autophagy related protein 7), and ceramide synthesis, as distinct from classical autophagy (Leidal and Debnath, 2021; Leidal et al., 2020). We propose that LC3B activation by CB2R is not only involved in intracellular membrane trafficking but also in intercellular signaling in the regulation of EV loading and secretion by lysosomal exocytosis, exosome release, and secretory autophagy (Leidal and Debnath, 2021; Liu et al., 2020b; Buratta et al., 2020). Secreted materials range from cytokines, lipids, and granules to virus particles. Secretory autophagy has been implicated in multiple diseases including cancer and neurodegeneration (New and Thomas, 2019). Pancreatic β-cells secrete insulin-containing EVs into the islet milieu that are recognized by the infiltrating dendritic cells and macrophages in NOD (non-obese diabetic) mice (Ferris et al., 2016), a mouse model of T1DM, resulting in the activation of APCs, which in turn with the help of MHC-II, are responsible for presenting insulin B-chain peptide and its fragments to reactive CD4^+^T cells (Vomund et al., 2015). Anti-inflammatory CB2R restrains M1 macrophage activation in the lean state (Wu et al., 2020b; Xu et al., 2013), however, it seems to lose this ability in obese states. Obese adipocytes are depleted of TFEB (Trivedi et al., 2016) and secrete more lipid-filled exosome-sized vesicles (AdExos) that are taken up by adipose tissue macrophages (ATMs) for triacylglyceride hydrolysis that then returns to adipocytes through macrophage presenting exosome-sized vesicles (MacExos) (Flaherty et al., 2019). The accumulation of lipofuscin in ATMs causes a switch from a lean M2 “alternatively activated” state to an obese M1 “classically activated” state generating a F4/80^+^CD11c^+^CD45hi dendritic cell subpopulation (Lumeng et al., 2007). CB2R activation reduces Iba1^+^ M1 population and increases the M2 population that might exert protective effects against the vicious lipid cycle between obese adipocytes and ATMs (Zarruk et al., 2012) and activation of TFEB due to dephosphorylation by calcineurin then activates autophagy-based hydrolysis of lipid droplets and protects against obesity-induced insulin resistance (Kim et al., 2021) (Figure 7). Global CB2R knockout mice have an obese phenotype; however, whether this is due to dendritic and macrophage CB2R deficiency is not yet known. In order to eventually answer this, we have created *Cnr2*-floxed mice that can be crossed with CX3CR1-Cre and CD11C-Cre mice to generate M1/M2-macrophages and F4/80^+^CD11c^+^ obesity-associated dendritic cell specific conditional CB2 knockout mice so that we can study macrophage activation and intercellular extracellular vesicle signaling and trafficking between adipocytes, β-cells and APCs (Liu et al., 2020a).

## Conclusion

The yin-yang relationship of CB1R and CB2R in pancreatic islets involves signaling *via* plasma membrane downstream signaling pathways and intra- and inter-cellular membranal trafficking. We propose that modulation of cannabinoid receptors will ameliorate T1DM by modulation of the mTORC/TFEB/calcineurin axis (Chiocco et al., 2010; Pan et al., 2020) and promotion of lysosome biogenesis that is a hub for T1DM tolerance, autophagy, and extracellular vesicle signaling. There is presently no cannabinoid therapeutic that increases the robustness of β-cells that can withstand the genetic lottery lost by people with pre-symptomatic and symptomatic T1DM. Cost effective, naturally occurring CB2R selective agonists widely used in traditional medicines and diets in Asia and South America for early intervention of diabetes are worthy of study in this regard. Additionally, next generation, selective, peripherally restricted synthetic cannabinoids that work by intervening in both CB1R and CB2R signaling are in the pipeline.

## References

[B1] Abioye A. Ayodele O. Marinkovic A. Patidar R. Akinwekomi A. Sanyaolu A. (2020). Δ9-Tetrahydrocannabivarin (THCV): a Commentary on Potential Therapeutic Benefit for the Management of Obesity and Diabetes. J. Cannabis Res. 2, 6. 10.1186/s42238-020-0016-7 33526143 PMC7819335

[B2] Agudo J. Martin M. Roca C. Molas M. Bura A. S. Zimmer A. (2010). Deficiency of CB2 Cannabinoid Receptor in Mice Improves Insulin Sensitivity but Increases Food Intake and Obesity with Age. Diabetologia 53, 2629–2640. 10.1007/s00125-010-1894-6 20835701

[B3] Alcigir M. E. Dogan H. O. Atalay Vural S. Yilmaz F. M. (2017). Neuroprotective Activity of Cannabinoid Receptor-2 against Oxidative Stress and Apoptosis in Rat Pups Having Experimentally-Induced Congenital Hypothyroidism. Dev. Neurobiol. 77, 1334–1347. 10.1002/dneu.22516 28799288

[B4] Alexandropoulos K. Bonito A. J. Weinstein E. G. Herbin O. (2015). Medullary Thymic Epithelial Cells and central Tolerance in Autoimmune Hepatitis Development: Novel Perspective from a New Mouse Model. Int. J. Mol. Sci. 16, 1980–2000. 10.3390/ijms16011980 25603179 PMC4307344

[B5] Alon R. Shulman Z. (2011). Chemokine Triggered Integrin Activation and Actin Remodeling Events Guiding Lymphocyte Migration across Vascular Barriers. Exp. Cel Res 317, 632–641. 10.1016/j.yexcr.2010.12.007 21376176

[B6] Alshaarawy O. Kurjan E. Truong N. Olson L. K. (2019). Diet-Induced Obesity in Cannabinoid-2 Receptor Knockout Mice and Cannabinoid Receptor 1/2 Double-Knockout Mice. Obesity (Silver Spring) 27, 454–461. 10.1002/oby.22403 30699233 PMC6429563

[B7] Annunziata P. Cioni C. Mugnaini C. Corelli F. (2017). Potent Immunomodulatory Activity of a Highly Selective Cannabinoid CB2 Agonist on Immune Cells from Healthy Subjects and Patients with Multiple Sclerosis. J. Neuroimmunol 303, 66–74. 10.1016/j.jneuroim.2016.12.009 28041663

[B8] Aseer K. R. Egan J. M. (2021). An Autonomous Cannabinoid System in Islets of Langerhans. Front. Endocrinol. (Lausanne) 12, 699661. 10.3389/fendo.2021.699661 34290671 PMC8287299

[B9] Basu S. Dittel B. N. (2011). Unraveling the Complexities of Cannabinoid Receptor 2 (CB2) Immune Regulation in Health and Disease. Immunol. Res. 51, 26–38. 10.1007/s12026-011-8210-5 21626285 PMC4624216

[B10] Behl T. Chadha S. Sachdeva M. Sehgal A. Kumar A. Venkatachalam T (2021). Understanding the Possible Role of Endocannabinoid System in Obesity. Prostaglandins Other Lipid Mediat 152, 106520. 10.1016/j.prostaglandins.2020.106520 33249225

[B11] Beji C. Loucif H. Telittchenko R. Olagnier D. Dagenais-Lussier X. van Grevenynghe J. (2020). Cannabinoid-Induced Immunomodulation during Viral Infections: A Focus on Mitochondria. Viruses 12, 875. 10.3390/v12080875 PMC7472050 32796517

[B12] Bellini G. Olivieri A. N. Grandone A. Alessio M. Gicchino M. F. Nobili B. (2015). Association between Cannabinoid Receptor Type 2 Q63R Variant and Oligo/polyarticular Juvenile Idiopathic Arthritis. Scand. J. Rheumatol. 44, 284–287. 10.3109/03009742.2015.1020863 25974389

[B13] Benner C. van der Meulen T. Cacéres E. Tigyi K. Donaldson C. J. Huising M. O. (2014). The Transcriptional Landscape of Mouse Beta Cells Compared to Human Beta Cells Reveals Notable Species Differences in Long Non-coding RNA and Protein-Coding Gene Expression. BMC Genomics 15, 620. 10.1186/1471-2164-15-620 25051960 PMC4124169

[B14] Bennett S. T. Lucassen A. M. Gough S. C. Powell E. E. Undlien D. E. Pritchard L. E. (1995). Susceptibility to Human Type 1 Diabetes at IDDM2 Is Determined by Tandem Repeat Variation at the Insulin Gene Minisatellite Locus. Nat. Genet. 9, 284–292. 10.1038/ng0395-284 7773291

[B15] Brailoiu G. C. Deliu E. Marcu J. Hoffman N. E. Console-Bram L. Zhao P. (2014). Differential Activation of Intracellular versus Plasmalemmal CB2 Cannabinoid Receptors. Biochemistry 53, 4990–4999. 10.1021/bi500632a 25033246 PMC4144709

[B16] Brailoiu G. C. Oprea T. I. Zhao P. Abood M. E. Brailoiu E. (2011). Intracellular Cannabinoid Type 1 (CB1) Receptors Are Activated by Anandamide. J. Biol. Chem. 286, 29166–29174. 10.1074/jbc.M110.217463 21719698 PMC3190724

[B17] Braun M. Khan Z. T. Khan M. B. Kumar M. Ward A. Achyut B. R. (2018). Selective Activation of Cannabinoid Receptor-2 Reduces Neuroinflammation after Traumatic Brain Injury via Alternative Macrophage Polarization. Brain Behav. Immun. 68, 224–237. 10.1016/j.bbi.2017.10.021 29079445 PMC5767553

[B18] Broca C. Varin E. Armanet M. Tourrel-Cuzin C. Bosco D. Dalle S. (2014). Proteasome Dysfunction Mediates High Glucose-Induced Apoptosis in Rodent Beta Cells and Human Islets. PLoS One 9, e92066. 10.1371/journal.pone.0092066 24642635 PMC3958412

[B19] Buckley N. E. McCoy K. L. Mezey E. Bonner T. Zimmer A. Felder C. C. (2000). Immunomodulation by Cannabinoids Is Absent in Mice Deficient for the Cannabinoid CB(2) Receptor. Eur. J. Pharmacol. 396, 141–149. 10.1016/s0014-2999(00)00211-9 10822068

[B20] Buratta S. Tancini B. Sagini K. Delo F. Chiaradia E. Urbanelli L. (2020). Lysosomal Exocytosis, Exosome Release and Secretory Autophagy: The Autophagic- and Endo-Lysosomal Systems Go Extracellular. Int. J. Mol. Sci. 21. 10.3390/ijms21072576 PMC7178086 32276321

[B21] Campanale C. Massarelli C. Savino I. Locaputo V. Uricchio V. F. (2020). A Detailed Review Study on Potential Effects of Microplastics and Additives of Concern on Human Health. Int. J. Environ. Res. Public Health 17. 10.3390/ijerph17041212 PMC7068600 32069998

[B22] Carayon P. Marchand J. Dussossoy D. Derocq J. M. Jbilo O. Bord A. (1998). Modulation and Functional Involvement of CB2 Peripheral Cannabinoid Receptors during B-Cell Differentiation. Blood 92, 3605–3615. 10.1182/blood.v92.10.3605.422k05_3605_3615 9808554

[B23] Carré A. Mallone R. (2021). Making Insulin and Staying Out of Autoimmune Trouble: The Beta-Cell Conundrum. Front. Immunol. 12, 639682. 10.3389/fimmu.2021.639682 33854508 PMC8039383

[B24] Cencioni M. T. Chiurchiù V. Catanzaro G. Borsellino G. Bernardi G. Battistini L. (2010). Anandamide Suppresses Proliferation and Cytokine Release from Primary Human T-Lymphocytes Mainly via CB2 Receptors. PLoS One 5, e8688. 10.1371/journal.pone.0008688 20098669 PMC2809084

[B25] Chen T. Xiong Y. Long M. Zheng D. Ke H. Xie J. (2019). Electro-acupuncture Pretreatment at Zusanli (ST36) Acupoint Attenuates Lipopolysaccharide-Induced Inflammation in Rats by Inhibiting Ca2+ Influx Associated with Cannabinoid CB2 Receptors. Inflammation 42, 211–220. 10.1007/s10753-018-0885-5 30168040

[B26] Chia C. Egan J. (2020). Incretins in Obesity and Diabetes. Ann. N. Y Acad. Sci. 1461, 104–126. 10.1111/nyas.14211 31392745 PMC10131087

[B27] Chiocco M. J. Zhu X. Walther D. Pletnikova O. Troncoso J. C. Uhl G. R. (2010). Fine Mapping of Calcineurin (PPP3CA) Gene Reveals Novel Alternative Splicing Patterns, Association of 5'UTR Trinucleotide Repeat with Addiction Vulnerability, and Differential Isoform Expression in Alzheimer's Disease. Subst. Use Misuse 45, 1809–1826. 10.3109/10826084.2010.482449 20590401 PMC3031160

[B28] Cinar R. Iyer M. R. Kunos G. (2020). The Therapeutic Potential of Second and Third Generation CB1R Antagonists. Pharmacol. Ther. 208, 107477. 10.1016/j.pharmthera.2020.107477 31926199 PMC8605822

[B29] Coopman K. Smith L. D. Wright K. L. Ward S. G. (2007). Temporal Variation in CB2R Levels Following T Lymphocyte Activation: Evidence that Cannabinoids Modulate CXCL12-Induced Chemotaxis. Int. Immunopharmacol 7, 360–371. 10.1016/j.intimp.2006.11.008 17276894

[B30] Cuervo A. M. (2011). Cell Biology. Autophagy's Top Chef. Science 332, 1392–1393. 10.1126/science.1208607 21680833

[B31] Dao M. François H. (2021). Cannabinoid Receptor 1 Inhibition in Chronic Kidney Disease: A New Therapeutic Toolbox. Front. Endocrinol. (Lausanne) 12, 720734. 10.3389/fendo.2021.720734 34305821 PMC8293381

[B32] Denaës T. Lodder J. Chobert M. N. Ruiz I. Pawlotsky J. M. Lotersztajn S. (2016). The Cannabinoid Receptor 2 Protects against Alcoholic Liver Disease via a Macrophage Autophagy-dependent Pathway. Sci. Rep. 6, 28806. 10.1038/srep28806 27346657 PMC4921859

[B33] Diedisheim M. Oshima M. Albagli O. Huldt C. W. Ahlstedt I. Clausen M. (2018). Modeling Human Pancreatic Beta Cell Dedifferentiation. Mol. Metab. 10, 74–86. 10.1016/j.molmet.2018.02.002 29472102 PMC5985229

[B34] Eisenbarth G. S. (1986). Type I Diabetes Mellitus. A Chronic Autoimmune Disease. N. Engl. J. Med. 314, 1360–1368. 10.1056/NEJM198605223142106 3517648

[B35] Eisenstein T. K. Meissler J. J. (2015). Effects of Cannabinoids on T-Cell Function and Resistance to Infection. J. Neuroimmune Pharmacol. 10, 204–216. 10.1007/s11481-015-9603-3 25876735 PMC4470840

[B36] Eizirik D. L. Colli M. L. Ortis F. (2009). The Role of Inflammation in Insulitis and Beta-Cell Loss in Type 1 Diabetes. Nat. Rev. Endocrinol. 5, 219–226. 10.1038/nrendo.2009.21 19352320

[B37] Elphick M. R. (2007). BfCBR: a Cannabinoid Receptor Ortholog in the Cephalochordate Branchiostoma floridae (Amphioxus). Gene 399, 65–71. 10.1016/j.gene.2007.04.025 17553639

[B38] Elphick M. R. (2002). Evolution of Cannabinoid Receptors in Vertebrates: Identification of a CB(2) Gene in the Puffer Fish Fugu Rubripes. Biol. Bull. 202, 104–107. 10.2307/1543648 11971807

[B39] Elphick M. R. Satou Y. Satoh N. (2003). The Invertebrate Ancestry of Endocannabinoid Signalling: an Orthologue of Vertebrate Cannabinoid Receptors in the Urochordate Ciona intestinalis. Gene 302, 95–101. 10.1016/s0378-1119(02)01094-6 12527200

[B40] Elphick M. R. (2012). The Evolution and Comparative Neurobiology of Endocannabinoid Signalling. Philos. Trans. R. Soc. Lond. B Biol. Sci. 367, 3201–3215. 10.1098/rstb.2011.0394 23108540 PMC3481536

[B41] Fan Y. Rudert W. A. Grupillo M. He J. Sisino G. Trucco M. (2009). Thymus-specific Deletion of Insulin Induces Autoimmune Diabetes. EMBO J. 28, 2812–2824. 10.1038/emboj.2009.212 19680229 PMC2750011

[B42] Fava A. Cimbro R. Wigley F. M. Liu Q. R. Rosen A. Boin F. (2016). Frequency of Circulating Topoisomerase-I-specific CD4 T Cells Predicts Presence and Progression of Interstitial Lung Disease in Scleroderma. Arthritis Res. Ther. 18, 99. 10.1186/s13075-016-0993-2 27145754 PMC4857293

[B43] Fernández-Ruiz J. Romero J. Velasco G. Tolón R. M. Ramos J. A. Guzmán M. (2007). Cannabinoid CB2 Receptor: a New Target for Controlling Neural Cell Survival? Trends Pharmacol. Sci. 28, 39–45. 10.1016/j.tips.2006.11.001 17141334

[B44] Ferris S. T. Carrero J. A. Unanue E. R. (2016). Antigen Presentation Events during the Initiation of Autoimmune Diabetes in the NOD Mouse. J. Autoimmun. 71, 19–25. 10.1016/j.jaut.2016.03.007 27021276 PMC4903912

[B45] Fierabracci A. (2014). The Putative Role of Proteolytic Pathways in the Pathogenesis of Type 1 Diabetes Mellitus: the 'autophagy' Hypothesis. Med. Hypotheses 82, 553–557. 10.1016/j.mehy.2014.02.010 24582332

[B46] Flaherty S. E. 3rd Grijalva A. Xu X. Ables E. Nomani A. Ferrante A. W. Jr. (2019). A Lipase-independent Pathway of Lipid Release and Immune Modulation by Adipocytes. Science 363, 989–993. 10.1126/science.aaw2586 30819964 PMC6579605

[B47] Flajnik M. F. Kasahara M. (2010). Origin and Evolution of the Adaptive Immune System: Genetic Events and Selective Pressures. Nat. Rev. Genet. 11, 47–59. 10.1038/nrg2703 19997068 PMC3805090

[B48] Galdino P. M. Nascimento M. V. Florentino I. F. Lino R. C. Fajemiroye J. O. Chaibub B. A. (2012). The Anxiolytic-like Effect of an Essential Oil Derived from Spiranthera Odoratissima A. St. Hil. Leaves and its Major Component, β-caryophyllene, in Male Mice. Prog. Neuropsychopharmacol. Biol. Psychiatry 38, 276–284. 10.1016/j.pnpbp.2012.04.012 22542869

[B49] Galiègue S. Mary S. Marchand J. Dussossoy D. Carrière D. Carayon P. (1995). Expression of central and Peripheral Cannabinoid Receptors in Human Immune Tissues and Leukocyte Subpopulations. Eur. J. Biochem. 232, 54–61. 10.1111/j.1432-1033.1995.tb20780.x 7556170

[B50] Geddo F. Scandiffio R. Antoniotti S. Cottone E. Querio G. Maffei M. E. (2019). PipeNig®-FL, a Fluid Extract of Black Pepper (Piper Nigrum L.) with a High Standardized Content of Trans-β-caryophyllene, Reduces Lipid Accumulation in 3T3-L1 Preadipocytes and Improves Glucose Uptake in C2C12 Myotubes. Nutrients 11, 2788. 10.3390/nu11112788 PMC6893583 31731718

[B51] Gerber P. A. Rutter G. A. (2017). The Role of Oxidative Stress and Hypoxia in Pancreatic Beta-Cell Dysfunction in Diabetes Mellitus. Antioxid. Redox Signal. 26, 501–518. 10.1089/ars.2016.6755 27225690 PMC5372767

[B52] Germic N. Frangez Z. Yousefi S. Simon H. U. (2019). Regulation of the Innate Immune System by Autophagy: Monocytes, Macrophages, Dendritic Cells and Antigen Presentation. Cell Death Differ 26, 715–727. 10.1038/s41418-019-0297-6 30737475 PMC6460400

[B53] González-Mariscal I. Krzysik-Walker S. M. Doyle M. E. Liu Q. R. Cimbro R. Santa-Cruz Calvo S. (2016). Human CB1 Receptor Isoforms, Present in Hepatocytes and β-cells, Are Involved in Regulating Metabolism. Sci. Rep. 6, 33302. 10.1038/srep33302 27641999 PMC5027555

[B54] González-Mariscal I. Montoro R. A. O'Connell J. F. Kim Y. Gonzalez-Freire M. Liu Q. R. (2019). Muscle Cannabinoid 1 Receptor Regulates Il-6 and Myostatin Expression, Governing Physical Performance and Whole-Body Metabolism. FASEB J. 33, 5850–5863. 10.1096/fj.201801145R 30726112 PMC6988864

[B55] Gonzalez-Mariscal I. Montoro R. A. Doyle M. E. Liu Q. R. Rouse M. O'Connell J. F. (2018). Absence of Cannabinoid 1 Receptor in Beta Cells Protects against High-Fat/high-Sugar Diet-Induced Beta Cell Dysfunction and Inflammation in Murine Islets. Diabetologia 61, 1470. 10.1007/s00125-018-4576-4 29497784 PMC6201315

[B56] Gorantla S. Makarov E. Roy D. Finke-Dwyer J. Murrin L. C. Gendelman H. E. (2010). Immunoregulation of a CB2 Receptor Agonist in a Murine Model of neuroAIDS. J. Neuroimmune Pharmacol. 5, 456–468. 10.1007/s11481-010-9225-8 20549374 PMC3109320

[B57] Grishman E. K. White P. C. Savani R. C. (2012). Toll-like Receptors, the NLRP3 Inflammasome, and Interleukin-1β in the Development and Progression of Type 1 Diabetes. Pediatr. Res. 71, 626–632. 10.1038/pr.2012.24 22337228

[B58] Group T. S. (2007). The Environmental Determinants of Diabetes in the Young (TEDDY) Study: Study Design. Pediatr. Diabetes 8, 286–298. 10.1111/j.1399-5448.2007.00269.x 17850472

[B59] Guha M. Saare M. Maslovskaja J. Kisand K. Liiv I. Haljasorg U. (2017). DNA Breaks and Chromatin Structural Changes Enhance the Transcription of Autoimmune Regulator Target Genes. J. Biol. Chem. 292, 6542–6554. 10.1074/jbc.M116.764704 28242760 PMC5399106

[B60] Gui H. Liu X. Wang Z. W. He D. Y. Su D. F. Dai S. M. (2014). Expression of Cannabinoid Receptor 2 and its Inhibitory Effects on Synovial Fibroblasts in Rheumatoid Arthritis. Rheumatology (Oxford) 53, 802–809. 10.1093/rheumatology/ket447 24440992

[B61] Guo K. Mou X. Huang J. Xiong N. Li H. (2014). Trans-caryophyllene Suppresses Hypoxia-Induced Neuroinflammatory Responses by Inhibiting NF-Κb Activation in Microglia. J. Mol. Neurosci. 54, 41–48. 10.1007/s12031-014-0243-5 24488604

[B62] Gupta M. Graham J. McNeeny B. Zarghami M. Landin-Olsson M. Hagopian W. A. (2006). MHC Class I Chain-Related Gene-A Is Associated with IA2 and IAA but Not GAD in Swedish Type 1 Diabetes Mellitus. Ann. N. Y Acad. Sci. 1079, 229–239. 10.1196/annals.1375.036 17130560

[B63] Han X. He Y. Bi G. H. Zhang H. Y. Song R. Liu Q. R. (2017). CB1 Receptor Activation on VgluT2-Expressing Glutamatergic Neurons Underlies Δ9-Tetrahydrocannabinol (Δ9-Thc)-Induced Aversive Effects in Mice. Sci. Rep. 7, 12315. 10.1038/s41598-017-12399-z 28951549 PMC5614984

[B64] Herold K. C. Bundy B. N. Long S. A. Bluestone J. A. DiMeglio L. A. Dufort M. J. (2019). An Anti-CD3 Antibody, Teplizumab, in Relatives at Risk for Type 1 Diabetes. N. Engl. J. Med. 381, 603–613. 10.1056/NEJMoa1902226 31180194 PMC6776880

[B65] Honkanen J. Nieminen J. K. Gao R. Luopajarvi K. Salo H. M. Ilonen J. (2010). IL-17 Immunity in Human Type 1 Diabetes. J. Immunol. 185, 1959–1967. 10.4049/jimmunol.1000788 20592279

[B66] Hu Y. Ranganathan M. Shu C. Liang X. Ganesh S. Osafo-Addo A. (2020). Single-cell Transcriptome Mapping Identifies Common and Cell-type Specific Genes Affected by Acute Delta9-tetrahydrocannabinol in Humans. Sci. Rep. 10, 3450. 10.1038/s41598-020-59827-1 32103029 PMC7044203

[B67] Hubbard-Lucey V. M. Shono Y. Maurer K. West M. L. Singer N. V. Ziegler C. G. (2014). Autophagy Gene Atg16L1 Prevents Lethal T Cell Alloreactivity Mediated by Dendritic Cells. Immunity 41, 579–591. 10.1016/j.immuni.2014.09.011 25308334 PMC4237219

[B68] Ilonen J. Lempainen J. Veijola R. (2019). The Heterogeneous Pathogenesis of Type 1 Diabetes Mellitus. Nat. Rev. Endocrinol. 15, 635–650. 10.1038/s41574-019-0254-y 31534209

[B69] Insel R. A. Dunne J. L. Atkinson M. A. Chiang J. L. Dabelea D. Gottlieb P. A. (2015). Staging Presymptomatic Type 1 Diabetes: a Scientific Statement of JDRF, the Endocrine Society, and the American Diabetes Association. Diabetes Care 38, 1964–1974. 10.2337/dc15-1419 26404926 PMC5321245

[B70] Ismail M. Khawaja G. (2018). Study of Cannabinoid Receptor 2 Q63R Gene Polymorphism in Lebanese Patients with Rheumatoid Arthritis. Clin. Rheumatol. 37, 2933–2938. 10.1007/s10067-018-4217-9 30032418

[B71] Jadoon K. A. Ratcliffe S. H. Barrett D. A. Thomas E. L. Stott C. Bell J. D. (2016). Efficacy and Safety of Cannabidiol and Tetrahydrocannabivarin on Glycemic and Lipid Parameters in Patients with Type 2 Diabetes: A Randomized, Double-Blind, Placebo-Controlled, Parallel Group Pilot Study. Diabetes Care 39, 1777–1786. 10.2337/dc16-0650 27573936

[B72] Jia W. He M. McLeod I. He Y. (2013). Autophagy, a Novel Pathway to Regulate Calcium Mobilization in T Lymphocytes. Front. Immunol. 4, 179. 10.3389/fimmu.2013.00179 23847620 PMC3701145

[B73] Jimenez-Blasco D. Busquets-Garcia A. Hebert-Chatelain E. Serrat R. Vicente-Gutierrez C. Ioannidou C. (2020). Glucose Metabolism Links Astroglial Mitochondria to Cannabinoid Effects. Nature 583, 603–608. 10.1038/s41586-020-2470-y 32641832

[B74] Jing N. Fang B. Li Z. Tian A. (2020). Exogenous Activation of Cannabinoid-2 Receptor Modulates TLR4/MMP9 Expression in a Spinal Cord Ischemia Reperfusion Rat Model. J. Neuroinflammation 17, 101. 10.1186/s12974-020-01784-7 32248810 PMC7132899

[B75] Joseph J. Niggemann B. Zaenker K. S. Entschladen F. (2004). Anandamide Is an Endogenous Inhibitor for the Migration of Tumor Cells and T Lymphocytes. Cancer Immunol. Immunother. 53, 723–728. 10.1007/s00262-004-0509-9 15034673 PMC11032818

[B76] Joshi N. Onaivi E. S. (2019). Endocannabinoid System Components: Overview and Tissue Distribution. Adv. Exp. Med. Biol. 1162, 1–12. 10.1007/978-3-030-21737-2_1 31332731

[B77] Jurewicz M. M. Stern L. J. (2019). Class II MHC Antigen Processing in Immune Tolerance and Inflammation. Immunogenetics 71, 171–187. 10.1007/s00251-018-1095-x 30421030 PMC6377339

[B78] Kapellos T. S. Recio C. Greaves D. R. Iqbal A. J. (2017). Cannabinoid Receptor 2 Modulates Neutrophil Recruitment in a Murine Model of Endotoxemia. Mediators Inflamm. 2017, 4315412. 10.1155/2017/4315412 28852269 PMC5567445

[B79] Kapellos T. S. Taylor L. Feuerborn A. Valaris S. Hussain M. T. Rainger G. E. (2019). Cannabinoid Receptor 2 Deficiency Exacerbates Inflammation and Neutrophil Recruitment. FASEB J. 33, 6154–6167. 10.1096/fj.201802524R 30799631 PMC6629158

[B80] Karaliota S. Siafaka-Kapadai A. Gontinou C. Psarra K. Mavri-Vavayanni M. (2009). Anandamide Increases the Differentiation of Rat Adipocytes and Causes PPARgamma and CB1 Receptor Upregulation. Obesity (Silver Spring) 17, 1830–1838. 10.1038/oby.2009.177 19543211

[B81] Karmaus P. W. Chen W. Crawford R. Kaplan B. L. Kaminski N. E. (2013). Δ9-tetrahydrocannabinol Impairs the Inflammatory Response to Influenza Infection: Role of Antigen-Presenting Cells and the Cannabinoid Receptors 1 and 2. Toxicol. Sci. 131, 419–433. 10.1093/toxsci/kfs315 23152191 PMC3551428

[B82] Kataoka K. Bilkei-Gorzo A. Nozaki C. Togo A. Nakamura K. Ohta K. (2020). Age-dependent Alteration in Mitochondrial Dynamics and Autophagy in Hippocampal Neuron of Cannabinoid CB1 Receptor-Deficient Mice. Brain Res. Bull. 160, 40–49. 10.1016/j.brainresbull.2020.03.014 32294520

[B83] Kaufmann U. Kahlfuss S. Yang J. Ivanova E. Koralov S. B. Feske S. (2019). Calcium Signaling Controls Pathogenic Th17 Cell-Mediated Inflammation by Regulating Mitochondrial Function. Cell Metab 29, 1104–e6. 10.1016/j.cmet.2019.01.019 30773462 PMC6506368

[B84] Ke P. Shao B. Z. Xu Z. Q. Wei W. Han B. Z. Chen X. W. (2016). Activation of Cannabinoid Receptor 2 Ameliorates DSS-Induced Colitis through Inhibiting NLRP3 Inflammasome in Macrophages. PLoS One 11, e0155076. 10.1371/journal.pone.0155076 27611972 PMC5017608

[B85] Kim J. Kim S. H. Kang H. Lee S. Park S. Y. Cho Y. (2021). TFEB-GDF15 axis Protects against Obesity and Insulin Resistance as a Lysosomal Stress Response. Nat. Metab. 3, 410–427. 10.1038/s42255-021-00368-w 33758420

[B86] Kim Y. Gautam S. Aseer K. R. Kim J. Chandrasekaran P. Mazucanti C. H. (2020). Hepatocyte Cannabinoid 1 Receptor Nullification Alleviates Toxin-Induced Liver Damage via NF-Κb Signaling. Cell Death Dis 11, 1044. 10.1038/s41419-020-03261-8 33298885 PMC7726564

[B87] King S. E. Skinner M. K. (2020). Epigenetic Transgenerational Inheritance of Obesity Susceptibility. Trends Endocrinol. Metab. 31, 478–494. 10.1016/j.tem.2020.02.009 32521235 PMC8260009

[B88] Knip M. Siljander H. (2016). The Role of the Intestinal Microbiota in Type 1 Diabetes Mellitus. Nat. Rev. Endocrinol. 12, 154–167. 10.1038/nrendo.2015.218 26729037

[B89] Kraj P. Ignatowicz L. (2018). The Mechanisms Shaping the Repertoire of CD4+ Foxp3+ Regulatory T Cells. Immunology 153, 290–296. 10.1111/imm.12859 29106696 PMC5795179

[B90] Lauterbach M. A. Wunderlich F. T. (2017). Macrophage Function in Obesity-Induced Inflammation and Insulin Resistance. Pflugers Arch. 469, 385–396. 10.1007/s00424-017-1955-5 28233125 PMC5362664

[B91] Leidal A. M. Debnath J. (2021). Emerging Roles for the Autophagy Machinery in Extracellular Vesicle Biogenesis and Secretion. FASEB Bioadv 3, 377–386. 10.1096/fba.2020-00138 33977236 PMC8103724

[B92] Leidal A. M. Huang H. H. Marsh T. Solvik T. Zhang D. Ye J. (2020). The LC3-Conjugation Machinery Specifies the Loading of RNA-Binding Proteins into Extracellular Vesicles. Nat. Cel Biol 22, 187–199. 10.1038/s41556-019-0450-y PMC7007875 31932738

[B93] Leinwand K. L. Jones A. A. Huang R. H. Jedlicka P. Kao D. J. de Zoeten E. F. (2017). Cannabinoid Receptor-2 Ameliorates Inflammation in Murine Model of Crohn's Disease. J. Crohns Colitis 11, 1369–1380. 10.1093/ecco-jcc/jjx096 28981653 PMC5881726

[B94] Levi D. Polychronakos C. (2013). Expression Profile of a Clonal Insulin-Expressing Epithelial Cell in the Thymus. Mol. Immunol. 56, 804–810. 10.1016/j.molimm.2013.07.015 23973805 PMC3792572

[B95] Li X. Hua T. Vemuri K. Ho J. H. Wu Y. Wu L. (2019). Crystal Structure of the Human Cannabinoid Receptor CB2. Cell 176, 459–e13. 10.1016/j.cell.2018.12.011 30639103 PMC6713262

[B96] Li Y. Li C. Li S. Peng Q. An N. A. He A. (2018). Human Exonization through Differential Nucleosome Occupancy. Proc. Natl. Acad. Sci. U S A. 115, 8817–8822. 10.1073/pnas.1802561115 30104384 PMC6126743

[B97] Littman D. R. Rudensky A. Y. (2010). Th17 and Regulatory T Cells in Mediating and Restraining Inflammation. Cell 140, 845–858. 10.1016/j.cell.2010.02.021 20303875

[B98] Liu Q. Wu Z. Liu Y. Chen L. Zhao H. Guo H. (2020). Cannabinoid Receptor 2 Activation Decreases Severity of Cyclophosphamide-Induced Cystitis via Regulating Autophagy. Neurourol Urodyn 39, 158–169. 10.1002/nau.24205 31729056

[B99] Liu Q. R. Canseco-Alba A. Zhang H. Y. Tagliaferro P. Chung M. Dennis E. (2017). Cannabinoid Type 2 Receptors in Dopamine Neurons Inhibits Psychomotor Behaviors, Alters Anxiety, Depression and Alcohol Preference. Sci. Rep. 7, 17410. 10.1038/s41598-017-17796-y 29234141 PMC5727179

[B100] Liu Q. R. Canseco-Alba A. Liang Y. Ishiguro H. Onaivi E. S. (2020). Low Basal CB2R in Dopamine Neurons and Microglia Influences Cannabinoid Tetrad Effects. Int. J. Mol. Sci. 21. 10.3390/ijms21249763 PMC7767340 33371336

[B101] Liu Q. R. Huang N. S. Qu H. O'Connell J. F. Gonzalez-Mariscal I. Santa-Cruz-Calvo S. (2019). Identification of Novel Mouse and Rat CB1R Isoforms and In Silico Modeling of Human CB1R for Peripheral Cannabinoid Therapeutics. Acta Pharmacol. Sin 40, 387–397. 10.1038/s41401-018-0152-1 30202012 PMC6460360

[B102] Liu Q. R. Pan C. H. Hishimoto A. Li C. Y. Xi Z. X. Llorente-Berzal A. (2009). Species Differences in Cannabinoid Receptor 2 (CNR2 Gene): Identification of Novel Human and Rodent CB2 Isoforms, Differential Tissue Expression and Regulation by Cannabinoid Receptor Ligands. Genes Brain Behav. 8, 519–530. 10.1111/j.1601-183X.2009.00498.x 19496827 PMC3389515

[B103] Liu W. Chen C. Gu X. Zhang L. Mao X. Chen Z. (2021). AM1241 Alleviates Myocardial Ischemia-Reperfusion Injury in Rats by Enhancing Pink1/Parkin-Mediated Autophagy. Life Sci. 272, 119228. 10.1016/j.lfs.2021.119228 33607150

[B104] Loi M. Müller A. Steinbach K. Niven J. Barreira da Silva R. Paul P. (2016). Macroautophagy Proteins Control MHC Class I Levels on Dendritic Cells and Shape Anti-viral CD8(+) T Cell Responses. Cell Rep 15, 1076–1087. 10.1016/j.celrep.2016.04.002 27117419

[B105] López A. Aparicio N. Pazos M. R. Grande M. T. Barreda-Manso M. A. Benito-Cuesta I. (2018). Cannabinoid CB2 Receptors in the Mouse Brain: Relevance for Alzheimer's Disease. J. Neuroinflammation 15, 158. 10.1186/s12974-018-1174-9 29793509 PMC5968596

[B106] Lu H. C. Mackie K. (2021). Review of the Endocannabinoid System. Biol. Psychiatry Cogn. Neurosci. Neuroimaging 6, 607–615. 10.1016/j.bpsc.2020.07.016 32980261 PMC7855189

[B107] Lumeng C. N. Bodzin J. L. Saltiel A. R. (2007). Obesity Induces a Phenotypic Switch in Adipose Tissue Macrophage Polarization. J. Clin. Invest. 117, 175–184. 10.1172/JCI29881 17200717 PMC1716210

[B108] Lundberg A. S. McDevitt H. O. (1992). Evolution of Major Histocompatibility Complex Class II Allelic Diversity: Direct Descent in Mice and Humans. Proc. Natl. Acad. Sci. U S A. 89, 6545–6549. 10.1073/pnas.89.14.6545 1631156 PMC49538

[B109] Lynes M. Kodani S. Tseng Y. (2019). Lipokines and Thermogenesis. Endocrinology 160, 2314–2325. 10.1210/en.2019-00337 31504387 PMC6760332

[B110] Mahmoud Gouda H. Mohamed Kamel N. R. (2013). Cannabinoid CB2 Receptor Gene (CNR2) Polymorphism Is Associated with Chronic Childhood Immune Thrombocytopenia in Egypt. Blood Coagul. Fibrinolysis 24, 247–251. 10.1097/MBC.0b013e32835aba1d 23406660

[B111] Maja Cigrovski Berkovic M. C. Bilic-Curcic I. Gradiser M. Herman-Mahecic D. Cigrovski V. Ivandic M. (2017). Are We Compensating for the Lack of Physical Activity in Our Diabetic Patients with Treatment Intensification? Sports (Basel) 5, 58. 10.3390/sports5030058 PMC5968955 29910418

[B112] Marasco M. R. Linnemann A. K. (2018). β-Cell Autophagy in Diabetes Pathogenesis. Endocrinology 159, 2127–2141. 10.1210/en.2017-03273 29617763 PMC5913620

[B113] Mariño E. Villanueva J. Walters S. Liuwantara D. Mackay F. Grey S. T. (2009). CD4(+)CD25(+) T-Cells Control Autoimmunity in the Absence of B-Cells. Diabetes 58, 1568–1577. 10.2337/db08-1504 19336675 PMC2699852

[B114] Marti-Solano M. Crilly S. E. Malinverni D. Munk C. Harris M. Pearce A. (2020). Combinatorial Expression of GPCR Isoforms Affects Signalling and Drug Responses. Nature 587, 650–656. 10.1038/s41586-020-2888-2 33149304 PMC7611127

[B115] Mathis D. Benoist C. (2009). Aire. Annu. Rev. Immunol. 27, 287–312. 10.1146/annurev.immunol.25.022106.141532 19302042

[B116] Matias I. McPartland J. M. Di Marzo V. (2005). Occurrence and Possible Biological Role of the Endocannabinoid System in the Sea Squirt Ciona intestinalis. J. Neurochem. 93, 1141–1156. 10.1111/j.1471-4159.2005.03103.x 15934935

[B117] McCoy K. L. Matveyeva M. Carlisle S. J. Cabral G. A. (1999). Cannabinoid Inhibition of the Processing of Intact Lysozyme by Macrophages: Evidence for CB2 Receptor Participation. J. Pharmacol. Exp. Ther. 289, 1620–1625. 10336560

[B118] McPartland J. M. Matias I. Di Marzo V. Glass M. (2006). Evolutionary Origins of the Endocannabinoid System. Gene 370, 64–74. 10.1016/j.gene.2005.11.004 16434153

[B119] Medina D. L. Di Paola S. Peluso I. Armani A. De Stefani D. Venditti R. (2015). Lysosomal Calcium Signalling Regulates Autophagy through Calcineurin and ​TFEB. Nat. Cel Biol 17, 288–299. 10.1038/ncb3114 PMC4801004 25720963

[B120] Mestre L. Correa F. Arévalo-Martín A. Molina-Holgado E. Valenti M. Ortar G. (2005). Pharmacological Modulation of the Endocannabinoid System in a Viral Model of Multiple Sclerosis. J. Neurochem. 92, 1327–1339. 10.1111/j.1471-4159.2004.02979.x 15748152

[B121] Michels A. Zhang L. Khadra A. Kushner J. A. Redondo M. J. Pietropaolo M. (2015). Prediction and Prevention of Type 1 Diabetes: Update on success of Prediction and Struggles at Prevention. Pediatr. Diabetes 16, 465–484. 10.1111/pedi.12299 26202050 PMC4592445

[B122] Muralidharan C. Conteh A. M. Marasco M. R. Crowder J. J. Kuipers J. de Boer P. (2021). Pancreatic Beta Cell Autophagy Is Impaired in Type 1 Diabetes. Diabetologia 64, 865–877. 10.1007/s00125-021-05387-6 33515072 PMC7940272

[B123] Nakahira K. Haspel J. A. Rathinam V. A. Lee S. J. Dolinay T. Lam H. C. (2011). Autophagy Proteins Regulate Innate Immune Responses by Inhibiting the Release of Mitochondrial DNA Mediated by the NALP3 Inflammasome. Nat. Immunol. 12, 222–230. 10.1038/ni.1980 21151103 PMC3079381

[B124] Nascimento M. V. Galdino P. M. Florentino I. F. de Brito A. F. Vanderlinde F. A. de Paula J. R. (2012). Anti-inflammatory Effect of Spiranthera Odoratissima A. St.-Hil. Leaves Involves Reduction of TNF-α. Nat. Prod. Res. 26, 2274–2279. 10.1080/14786419.2011.653973 22292909

[B125] Navarrete C. Carrillo-Salinas F. Palomares B. Mecha M. Jiménez-Jiménez C. Mestre L. (2018). Hypoxia Mimetic Activity of VCE-004.8, a Cannabidiol Quinone Derivative: Implications for Multiple Sclerosis Therapy. J. Neuroinflammation 15, 64. 10.1186/s12974-018-1103-y 29495967 PMC5831753

[B126] New J. Thomas S. M. (2019). Autophagy-dependent Secretion: Mechanism, Factors Secreted, and Disease Implications. Autophagy 15, 1682–1693. 10.1080/15548627.2019.1596479 30894055 PMC6735501

[B127] Newsholme P. Keane K. N. Carlessi R. Cruzat V. (2019). Oxidative Stress Pathways in Pancreatic β-cells and Insulin-Sensitive Cells and Tissues: Importance to Cell Metabolism, Function, and Dysfunction. Am. J. Physiol. Cel Physiol 317, C420–C433. 10.1152/ajpcell.00141.2019 31216193

[B128] Nielsen J. E. Rolland A. D. Rajpert-De Meyts E. Janfelt C. Jørgensen A. Winge S. B. (2019). Characterisation and Localisation of the Endocannabinoid System Components in the Adult Human Testis. Sci. Rep. 9, 12866. 10.1038/s41598-019-49177-y 31537814 PMC6753062

[B129] Noble J. A. Valdes A. M. (2011). Genetics of the HLA Region in the Prediction of Type 1 Diabetes. Curr. Diab Rep. 11, 533–542. 10.1007/s11892-011-0223-x 21912932 PMC3233362

[B130] Øynebråten I. (2020). Involvement of Autophagy in MHC Class I Antigen Presentation. Scand. J. Immunol. 92, e12978. 10.1111/sji.12978 32969499 PMC7685157

[B131] Pacher P. Kogan N. M. Mechoulam R. (2020). Beyond THC and Endocannabinoids. Annu. Rev. Pharmacol. Toxicol. 60, 637–659. 10.1146/annurev-pharmtox-010818-021441 31580774

[B132] Palazuelos J. Davoust N. Julien B. Hatterer E. Aguado T. Mechoulam R. (2008). The CB(2) Cannabinoid Receptor Controls Myeloid Progenitor Trafficking: Involvement in the Pathogenesis of an Animal Model of Multiple Sclerosis. J. Biol. Chem. 283, 13320–13329. 10.1074/jbc.M707960200 18334483

[B133] Pan B. Li J. Parajuli N. Tian Z. Wu P. Lewno M. T. (2020). The Calcineurin-TFEB-P62 Pathway Mediates the Activation of Cardiac Macroautophagy by Proteasomal Malfunction. Circ. Res. 127, 502–518. 10.1161/CIRCRESAHA.119.316007 32366200 PMC7416491

[B134] Parzych K. R. Klionsky D. J. (2014). An Overview of Autophagy: Morphology, Mechanism, and Regulation. Antioxid. Redox Signal. 20, 460–473. 10.1089/ars.2013.5371 23725295 PMC3894687

[B135] Patterson C. C. Karuranga S. Salpea P. Saeedi P. Dahlquist G. Soltesz G. (2019). Worldwide Estimates of Incidence, Prevalence and Mortality of Type 1 Diabetes in Children and Adolescents: Results from the International Diabetes Federation Diabetes Atlas, 9th Edition. Diabetes Res. Clin. Pract. 157, 107842. 10.1016/j.diabres.2019.107842 31518658

[B136] Pellati F. Brighenti V. Sperlea J. Marchetti L. Bertelli D. Benvenuti S. (2018). New Methods for the Comprehensive Analysis of Bioactive Compounds in Cannabis Sativa L. (Hemp). Molecules 23, 2639. 10.3390/molecules23102639 PMC6222702 30322208

[B137] Peng W. Wong Y. C. Krainc D. (2020). Mitochondria-lysosome Contacts Regulate Mitochondrial Ca2+ Dynamics via Lysosomal TRPML1. Proc. Natl. Acad. Sci. U S A. 117, 19266–19275. 10.1073/pnas.2003236117 32703809 PMC7430993

[B138] Polsky S. Ellis S. L. (2015). Obesity, Insulin Resistance, and Type 1 Diabetes Mellitus. Curr. Opin. Endocrinol. Diabetes Obes. 22, 277–282. 10.1097/MED.0000000000000170 26087341

[B139] Rezq S. Hassan R. Mahmoud M. F. (2021). Rimonabant Ameliorates Hepatic Ischemia/reperfusion Injury in Rats: Involvement of Autophagy via Modulating ERK- and PI3K/AKT-mTOR Pathways. Int. Immunopharmacol 100, 108140. 10.1016/j.intimp.2021.108140 34536742

[B140] Rieder S. A. Chauhan A. Singh U. Nagarkatti M. Nagarkatti P. (2010). Cannabinoid-induced Apoptosis in Immune Cells as a Pathway to Immunosuppression. Immunobiology 215, 598–605. 10.1016/j.imbio.2009.04.001 19457575 PMC3005548

[B141] Roche P. A. Furuta K. (2015). The Ins and Outs of MHC Class II-Mediated Antigen Processing and Presentation. Nat. Rev. Immunol. 15, 203–216. 10.1038/nri3818 25720354 PMC6314495

[B142] Rogers M. A. M. Kim C. Banerjee T. Lee J. M. (2017). Fluctuations in the Incidence of Type 1 Diabetes in the United States from 2001 to 2015: a Longitudinal Study. BMC Med. 15, 199. 10.1186/s12916-017-0958-6 29115947 PMC5688827

[B143] Rom S. Zuluaga-Ramirez V. Dykstra H. Reichenbach N. L. Pacher P. Persidsky Y. (2013). Selective Activation of Cannabinoid Receptor 2 in Leukocytes Suppresses Their Engagement of the Brain Endothelium and Protects the Blood-Brain Barrier. Am. J. Pathol. 183, 1548–1558. 10.1016/j.ajpath.2013.07.033 24055259 PMC3814716

[B144] Rossi F. Bellini G. Tolone C. Luongo L. Mancusi S. Papparella A. (2012). The Cannabinoid Receptor Type 2 Q63R Variant Increases the Risk of Celiac Disease: Implication for a Novel Molecular Biomarker and Future Therapeutic Intervention. Pharmacol. Res. 66, 88–94. 10.1016/j.phrs.2012.03.011 22465144

[B145] Sam A. H. Salem V. Ghatei M. A. (2011). Rimonabant: From RIO to Ban. J. Obes. 2011, 432607. 10.1155/2011/432607 21773005 PMC3136184

[B146] Schatz A. R. Lee M. Condie R. B. Pulaski J. T. Kaminski N. E. (1997). Cannabinoid Receptors CB1 and CB2: a Characterization of Expression and Adenylate Cyclase Modulation within the Immune System. Toxicol. Appl. Pharmacol. 142, 278–287. 10.1006/taap.1996.8034 9070350

[B147] Segerstolpe Å. Palasantza A. Eliasson P. Andersson E. M. Andréasson A. C. Sun X. (2016). Single-Cell Transcriptome Profiling of Human Pancreatic Islets in Health and Type 2 Diabetes. Cel Metab 24, 593–607. 10.1016/j.cmet.2016.08.020 PMC5069352 27667667

[B148] Settembre C. Di Malta C. Polito V. A. Garcia Arencibia M. Vetrini F. Erdin S. (2011). TFEB Links Autophagy to Lysosomal Biogenesis. Science 332, 1429–1433. 10.1126/science.1204592 21617040 PMC3638014

[B149] Shao B. Z. Wei W. Ke P. Xu Z. Q. Zhou J. X. Liu C. (2014). Activating Cannabinoid Receptor 2 Alleviates Pathogenesis of Experimental Autoimmune Encephalomyelitis via Activation of Autophagy and Inhibiting NLRP3 Inflammasome. CNS Neurosci. Ther. 20, 1021–1028. 10.1111/cns.12349 25417929 PMC6492996

[B150] Shao Z. Yin J. Chapman K. Grzemska M. Clark L. Wang J. (2016). High-resolution crystal Structure of the Human CB1 Cannabinoid Receptor. Nature 540, 602. 10.1038/nature20613 27851727 PMC5433929

[B151] Siani A. C. Souza M. C. Henriques M. G. Ramos M. F. (2013). Anti-inflammatory Activity of Essential Oils from Syzygium Cumini and Psidium Guajava. Pharm. Biol. 51, 881–887. 10.3109/13880209.2013.768675 23577801

[B152] Spence A. Purtha W. Tam J. Dong S. Kim Y. Ju C. H. (2018). Revealing the Specificity of Regulatory T Cells in Murine Autoimmune Diabetes. Proc. Natl. Acad. Sci. U S A. 115, 5265–5270. 10.1073/pnas.1715590115 29712852 PMC5960284

[B153] Stempel A. V. Stumpf A. Zhang H. Y. Özdoğan T. Pannasch U. Theis A. K. (2016). Cannabinoid Type 2 Receptors Mediate a Cell Type-specific Plasticity in the Hippocampus. Neuron 90, 795–809. 10.1016/j.neuron.2016.03.034 27133464 PMC5533103

[B154] Strehl B. Seifert U. Krüger E. Heink S. Kuckelkorn U. Kloetzel P. M. (2005). Interferon-gamma, the Functional Plasticity of the Ubiquitin-Proteasome System, and MHC Class I Antigen Processing. Immunol. Rev. 207, 19–30. 10.1111/j.0105-2896.2005.00308.x 16181324

[B155] Strisciuglio C. Bellini G. Miele E. Martinelli M. Cenni S. Tortora C. (2018). Cannabinoid Receptor 2 Functional Variant Contributes to the Risk for Pediatric Inflammatory Bowel Disease. J. Clin. Gastroenterol. 52, e37–e43. 10.1097/MCG.0000000000000755 27875353

[B156] Stumpf A. Parthier D. Sammons R. P. Stempel A. V. Breustedt J. Rost B. R. (2018). Cannabinoid Type 2 Receptors Mediate a Cell Type-specific Self-Inhibition in Cortical Neurons. Neuropharmacology 139, 217–225. 10.1016/j.neuropharm.2018.07.020 30025920

[B157] Su T. F. Zhao Y. Q. Zhang L. H. Peng M. Wu C. H. Pei L. (2012). Electroacupuncture Reduces the Expression of Proinflammatory Cytokines in Inflamed Skin Tissues through Activation of Cannabinoid CB2 Receptors. Eur. J. Pain 16, 624–635. 10.1002/j.1532-2149.2011.00055.x 22337285

[B158] Theofilopoulos A. N. Kono D. H. Baccala R. (2017). The Multiple Pathways to Autoimmunity. Nat. Immunol. 18, 716–724. 10.1038/ni.3731 28632714 PMC5791156

[B159] Tortora C. Punzo F. Argenziano M. Di Paola A. Tolone C. Strisciuglio C. (2020). The Role of Cannabinoid Receptor Type 2 in the Bone Loss Associated with Pediatric Celiac Disease. J. Pediatr. Gastroenterol. Nutr. 71, 633–640. 10.1097/MPG.0000000000002863 33093370

[B160] Trivedi P. C. Bartlett J. J. Perez L. J. Brunt K. R. Legare J. F. Hassan A. (2016). Glucolipotoxicity Diminishes Cardiomyocyte TFEB and Inhibits Lysosomal Autophagy during Obesity and Diabetes. Biochim. Biophys. Acta 1861, 1893–1910. 10.1016/j.bbalip.2016.09.004 27620487

[B161] Turcotte C. Blanchet M. R. Laviolette M. Flamand N. (2016). The CB2 Receptor and its Role as a Regulator of Inflammation. Cell Mol Life Sci 73, 4449–4470. 10.1007/s00018-016-2300-4 27402121 PMC5075023

[B162] Valecka J. Almeida C. R. Su B. Pierre P. Gatti E. (2018). Autophagy and MHC-Restricted Antigen Presentation. Mol. Immunol. 99, 163–170. 10.1016/j.molimm.2018.05.009 29787980

[B163] Vivot K. Pasquier A. Goginashvili A. Ricci R. (2020). Breaking Bad and Breaking Good: β-Cell Autophagy Pathways in Diabetes. J. Mol. Biol. 432, 1494–1513. 10.1016/j.jmb.2019.07.030 31381897

[B164] Vomund A. N. Zinselmeyer B. H. Hughes J. Calderon B. Valderrama C. Ferris S. T. (2015). Beta Cells Transfer Vesicles Containing Insulin to Phagocytes for Presentation to T Cells. Proc. Natl. Acad. Sci. U S A. 112, E5496–E5502. 10.1073/pnas.1515954112 26324934 PMC4603448

[B165] Wu A. Hu P. Lin J. Xia W. Zhang R. (2018). Activating Cannabinoid Receptor 2 Protects against Diabetic Cardiomyopathy through Autophagy Induction. Front. Pharmacol. 9, 1292. 10.3389/fphar.2018.01292 30459625 PMC6232417

[B166] Wu Q. Ma Y. Liu Y. Wang N. Zhao X. Wen D. (2020). CB2R Agonist JWH-133 Attenuates Chronic Inflammation by Restraining M1 Macrophage Polarization via Nrf2/HO-1 Pathway in Diet-Induced Obese Mice. Life Sci. 260, 118424. 10.1016/j.lfs.2020.118424 32949586

[B167] Wu Q. Zhang M. Liu X. Zhang J. Wang H. (2020). CB2R Orchestrates Neuronal Autophagy through Regulation of the mTOR Signaling Pathway in the hippocampus of Developing Rats with Status Epilepticus. Int. J. Mol. Med. 45, 475–484. 10.3892/ijmm.2019.4439 31894322 PMC6984801

[B168] Wu Y. Zhang N. Hashimoto K. Xia C. Dijkstra J. M. (2021). Structural Comparison between MHC Classes I and II; in Evolution, a Class-II-like Molecule Probably Came First. Front. Immunol. 12, 621153. 10.3389/fimmu.2021.621153 34194421 PMC8236899

[B169] Xi Z. X. Peng X. Q. Li X. Song R. Zhang H. Y. Liu Q. R. (2011). Brain Cannabinoid CB₂ Receptors Modulate Cocaine's Actions in Mice. Nat. Neurosci. 14, 1160–1166. 10.1038/nn.2874 21785434 PMC3164946

[B170] Xin Y. Kim J. Okamoto H. Ni M. Wei Y. Adler C. (2016). RNA Sequencing of Single Human Islet Cells Reveals Type 2 Diabetes Genes. Cel Metab 24, 608–615. 10.1016/j.cmet.2016.08.018 27667665

[B171] Xu A. Yang Y. Shao Y. Wu M. Sun Y. (2020). Activation of Cannabinoid Receptor Type 2-induced Osteogenic Differentiation Involves Autophagy Induction and P62-Mediated Nrf2 Deactivation. Cell Commun Signal 18, 9. 10.1186/s12964-020-0512-6 31941496 PMC6964093

[B172] Xu X. Grijalva A. Skowronski A. van Eijk M. Serlie M. J. Ferrante A. W. Jr. (2013). Obesity Activates a Program of Lysosomal-dependent Lipid Metabolism in Adipose Tissue Macrophages Independently of Classic Activation. Cel Metab 18, 816–830. 10.1016/j.cmet.2013.11.001 PMC3939841 24315368

[B173] Yedidi R. S. Wendler P. Enenkel C. (2017). AAA-ATPases in Protein Degradation. Front. Mol. Biosci. 4, 42. 10.3389/fmolb.2017.00042 28676851 PMC5476697

[B174] Yu S. J. Reiner D. Shen H. Wu K. J. Liu Q. R. Wang Y. (2015). Time-Dependent Protection of CB2 Receptor Agonist in Stroke. PLoS One 10, e0132487. 10.1371/journal.pone.0132487 26186541 PMC4505877

[B175] Zarruk J. G. Fernández-López D. García-Yébenes I. García-Gutiérrez M. S. Vivancos J. Nombela F. (2012). Cannabinoid Type 2 Receptor Activation Downregulates Stroke-Induced Classic and Alternative Brain Macrophage/microglial Activation Concomitant to Neuroprotection. Stroke 43, 211–219. 10.1161/STROKEAHA.111.631044 22020035

[B176] Zhang H. Y. Bi G. H. Li X. Li J. Qu H. Zhang S. J. (2015). Species Differences in Cannabinoid Receptor 2 and Receptor Responses to Cocaine Self-Administration in Mice and Rats. Neuropsychopharmacology 40, 1037–1051. 10.1038/npp.2014.297 25374096 PMC4330519

[B177] Zhang H. Y. Gao M. Shen H. Bi G. H. Yang H. J. Liu Q. R. (2017). Expression of Functional Cannabinoid CB2 Receptor in VTA Dopamine Neurons in Rats. Addict. Biol. 22, 752–765. 10.1111/adb.12367 26833913 PMC4969232

[B178] Zhang S. J. Wang C. Yan S. Fu A. Luan X. Li Y. (2017). Isoform Evolution in Primates through Independent Combination of Alternative RNA Processing Events. Mol. Biol. Evol. 34, 2453–2468. 10.1093/molbev/msx212 28957512 PMC5850651

[B179] Zheng X. Sun T. Wang X. (2013). Activation of Type 2 Cannabinoid Receptors (CB2R) Promotes Fatty Acid Oxidation through the SIRT1/PGC-1α Pathway. Biochem. Biophys. Res. Commun. 436, 377–381. 10.1016/j.bbrc.2013.05.108 23747418

[B180] Zimmer A. Zimmer A. M. Hohmann A. G. Herkenham M. Bonner T. I. (1999). Increased Mortality, Hypoactivity, and Hypoalgesia in Cannabinoid CB1 Receptor Knockout Mice. Proc. Natl. Acad. Sci. U S A. 96, 5780–5785. 10.1073/pnas.96.10.5780 10318961 PMC21937

[B181] Zucchelli S. Holler P. Yamagata T. Roy M. Benoist C. Mathis D. (2005). Defective central Tolerance Induction in NOD Mice: Genomics and Genetics. Immunity 22, 385–396. 10.1016/j.immuni.2005.01.015 15780994

